# Electrostatic Gating of Phosphorene Polymorphs

**DOI:** 10.1021/acs.jpcc.3c05876

**Published:** 2024-02-09

**Authors:** Fereshteh Mahmoodpouri Malayee, Robabeh Bagheri, Fariba Nazari, Francesc Illas

**Affiliations:** † Department of Chemistry, Institute for Advanced Studies in Basic Sciences, Zanjan 45137-66731, Iran; ‡ Center of Climate Change and Global Warming, Institute for Advanced Studies in Basic Sciences, Zanjan 45137-66731, Iran; § Departament de Ciència de Materials i Química Física & Institut de Química Teòrica i Computacional (IQTCUB), Universitat de Barcelona,C/Martí i Franquès 1, 08028 Barcelona, Spain

## Abstract

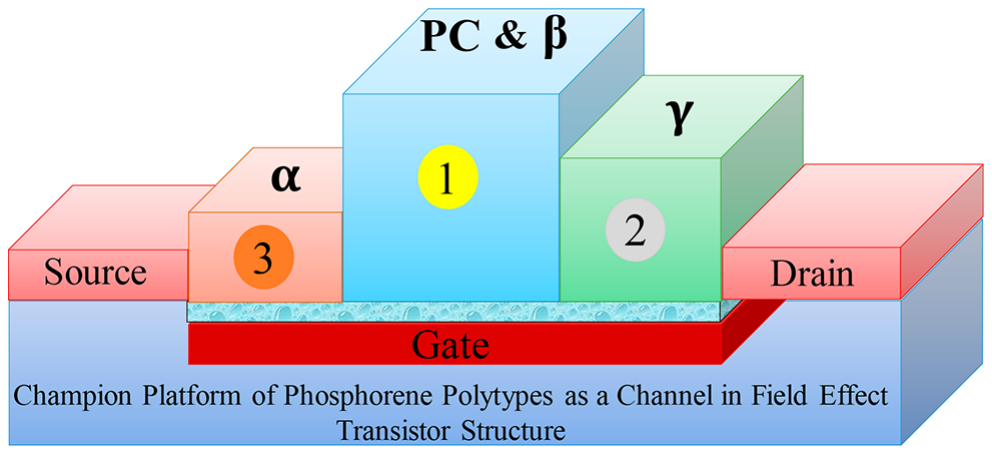

The ability to directly monitor the states of electrons in modern field-effect transistors (FETs) could transform our understanding of the physics and improve the function of related devices. In particular, phosphorene allotropes present a fertile landscape for the development of high-performance FETs. Using density functional theory-based methods, we have systematically investigated the influence of electrostatic gating on the structures, stabilities, and fundamental electronic properties of pristine and carbon-doped monolayer (bilayer) phosphorene allotropes. The remarkable flexibility of phosphorene allotropes, arising from intra- and interlayer van der Waals interactions, causes a good resilience up to equivalent gate potential of two electrons per unit cell. The resilience depends on the stacking details in such a way that rotated bilayers show considerably higher thermodynamical stability than the unrotated ones, even at a high gate potential. In addition, a semiconductor to metal phase transition is observed in some of the rotated and carbon-doped structures with increased electronic transport relative to graphene in the context of real space Green’s function formalism.

## 1 Introduction

The phenomenon of polytypism, a variant of polymorphism, is ubiquitous in layered materials.^1,2^ Polytypes exhibit the same close-packed planes but with a different stacking sequence in the third dimension, the one perpendicular to these planes. Hence, a diverse crystal structure can be obtained by just changing the layer stacking sequence, while the periodic structure of each layer is preserved. In fact, only the periodicity along the growth axis varies from one polytype to another. Polytypes of a material have analogous structural features as well as nearly the same internal energies, the simultaneous occurrence of several similar structures under identical growth conditions being at the heart of this phenomenon.^1,2^ Layered materials also exhibit other interesting properties and, in particular, have the potential for the development of high-performance field-effect transistors (FETs).^3^

Among layered materials, phosphorene is a promising candidate due to rich geometric structures that is leading to various polymorphs and polytypes^4−27^ while having none of the obstacles exhibited by other two-dimensional materials.^28−31^ Several structurally different two-dimensional polymorphs of phosphorus namely, α-P, β-P, γ-P, δ-P, ε-P, τ-P, η-P, θ-P, σ-P, ϕ-P, tricycle-type red phosphorene (R-P), square-octagon phosphorene (O–P) and hexagonal-star phosphorene (H–P) have been investigated using first principle methods.^32−34^ Most of these two-dimensional polymorphic materials are semiconductors with a band gap in the range of 0.4 to 2.1 eV.^7,35−37^ We recall that α-P and β-P have been prepared experimentally, while other allotropes have not yet been synthesized.^38^ According to the polytypism definition, the individual phosphorene allotropes can be coupled through noncovalent interactions leading to bi/tri/few layer phases up to the bulk material.^39^ The resulting stacks can be characterized into three types: in-plane shifted, in-plane twisted, and hybrids of the former two polymorphs. In addition, a new family of 2D materials consisting of phosphorus and carbon (phosphorene carbide, (PC)) has been rather recently predicted theoretically.^40−43^ Interestingly, the experimental realization of 2D PC allotropes was achieved by combining the theoretical predictions and previous experimental observations.^44^ These studies demonstrated that various structures in phosphorus carbide can be produced by considering different atomic ratios of P/C.^43−46^ Furthermore, structure, stability and electronic properties of PC monolayers, namely α-, β-, and γ-PC, have been predicted.^45^ Rather recently, Kou et al.^47^ suggested that polytypism and polymorphism in phosphorene play a crucial rule and provide a fertile landscape to design novel architectures and instructing new functionalities.

**Figure 1:**
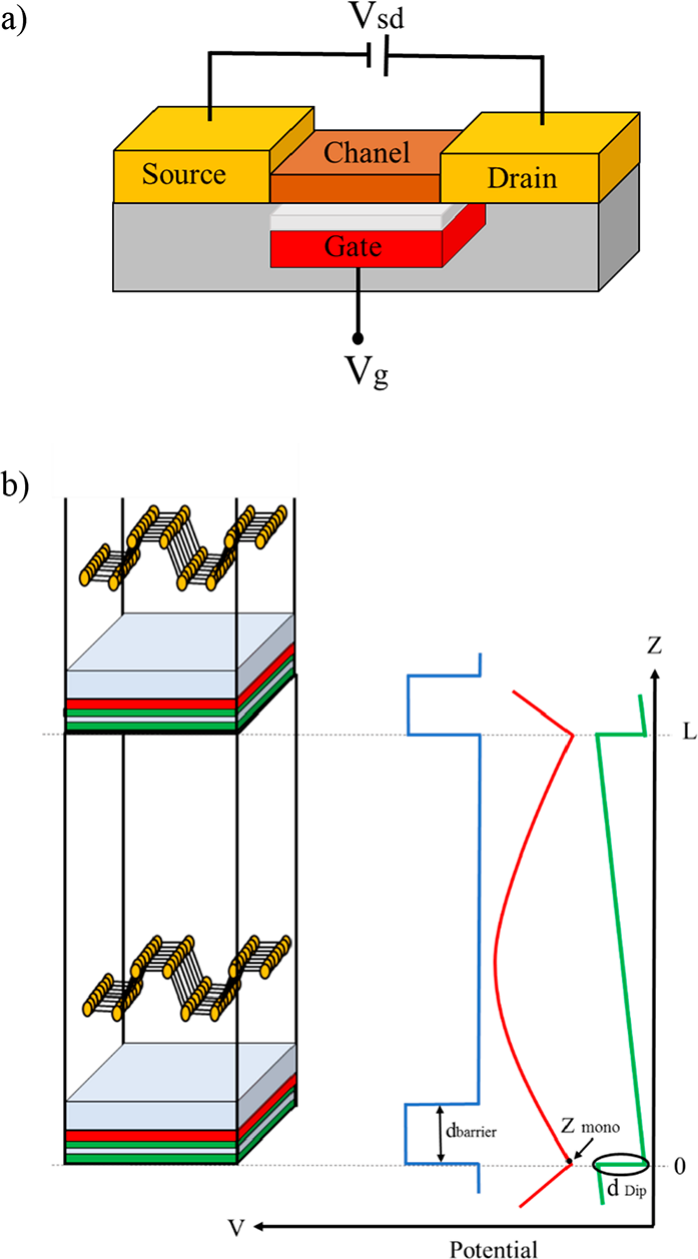
(a) Schematic representation of the Field Effect Transistor model used in the present work. (b) Sketch of a gated system model in a periodically repeated unit cell (left), potential energy barrier (blue), monopole potential (red), and dipole potential (green) in the *Z* direction.

On the other way, field-effect transistors revolutionized the field of electronics, enabling the development of smaller, faster, and more efficient devices. FETs work by controlling the flow of current through a semiconductor channel using an electric field, making the device more efficient and less prone to noise, which is important in high-speed and low-power applications. FETs consists of a gate (metal), a channel, a source, and a drain, all of which are made of a given semiconductor material (Figure 1a). The gate is separated from the semiconductor channel by a thin layer of an insulating material. When a voltage is applied to the gate, an electric field is created in the channel, which controls the flow of electrons between the source and the drain. The channel of an FET is a key component that determines the device electrical characteristics and performance. Recent research on FET channel materials has focused on improving device performance by optimizing the channel properties and exploring new materials and structures.^48−51^

The channel consists of a region of a semiconductor material connecting the source and drain terminals of the transistor. It is the pathway through which the current flows when the transistor is turned on. The conductivity of the channel can be controlled by varying the voltage on the gate terminal. The length and width of the channel also affect the transistor performance. A shorter channel allows for faster switching speeds but can lead to an increased leakage current and reduced reliability. A wider channel can handle higher currents but may also have a higher capacitance, which can limit the device performance under high frequency. Summing up, FETs have their own unique properties and applications, but the channel constitutes a fundamental component to controlling the current flow.^52−54^ The choice of semiconductor material depends on the specific application requirements, such as the operating frequency, power handling capability, and thermal performance. Recent studies have reported high-performance 2D- based FETs with record-high transconductance and low power consumption, and with high on/off ratio and sensitivity to light and gas molecules.^55−58^

There is evidence that field-effect transistors with p-channel such as phosphorene show higher performance and efficiency than other nanomaterials.^59^ This is confirmed by Das et al.^60^ who showed that in phosphorene the on-to-off current ratio (*I*_ON_/*I*_OF_) of holes (10^5^) is much larger than that of electrons (10^3^), which is very desirable in FETs. With this perspective, we have examined the capability of phosphorene polymorphs as channel candidates in the field-effect transistor and analyzed their geometry and electronic properties under the influence of the gate potential. Hereby, we report a first-principles study that clarifies the microscopic origin of the band gap variation in the electrostatic gated polytypes and polymorphs of phosphorene. In addition, we report transport properties of these structures in the context of real space Green’s function formalism that have prominent importance for FET’s technology.

## 2 Computational Details and Materials Models

Calculations reported in the present work have been carried out in the framework of density functional theory (DFT) using periodic models, as described below and in the next section. Due to our objective of comparing the trends in structural parameters and electronic properties, we utilized the cost-effective Perdew–Burke–Ernzerhof (PBE)^61^ forms of the generalized gradient approximation (GGA) exchange–correlation functional.^62^ However, to investigate whether changing the functional affects the trend of variations, we also employed the SCAN^63,64^ functional. Notably, the band gap obtained from the SCAN functional showed significant similarities to the band gap results corrected by meta-GGA, confirming our choice.

The electron density is expanded in plane-wave basis sets with a cutoff energy of 52 Ry and the interaction between the valence electrons and ion cores is described through the P.pbe-n-rrkjus_psl.1.0.0.UPF, C.pbe-n-rrkjus_psl.1.0.0.UPF ultrasoft pseudopotentials.^65^ The effect of van der Waals interactions is taken into account by the DFT-D3 approach due to Grimme et al.^66^ To avoid the interaction between the monolayer (bilayer) structure and its periodically repeated images in the slab model, a vacuum layer of 30 Å thickness is added. For all periodic systems, the Brillouin zone is sampled with 4 × 4 × 1 and 15 × 15 × 1 **k**-point meshes for the structural and electronic properties, respectively. It should be noticed that after optimizing the required number of **k** points along various directions while considering optimal conditions, we generated a uniform density of **k** points. The lattice constants and the positions of the atoms are optimized until the Hellmann–Feynman forces are less than 0.001 eV/Å. All calculations were carried out using the Quantum-Espresso package version qe-6.5^67^ and the XCrySDen^68^ and Vesta packages^69^ were used to visualize the atomic structures.

Using the experimental lattice parameter that is reported for black phosphorus (BP)^70,71^ we have designed the starting geometry of the unit cell of the different phosphorene polymorphs. We have classified the studied polymorphs into five categories: monolayer structures, polytypes, hybrid-polytypes, rotated polytypes, and PC. Information of the individual phosphorene allotropes are summarized in Table S1 and Figure S1a in the Supporting Information. We must point out that to ensure a meaningful comparison; a single cell with the same number of atoms (4 atoms) has been selected for all three starting structures (α, β, and γ), and among possible stable stackings, three high symmetry polytypes of AA, AB, and AC of α, β, and γ allotropes have been considered (Figure S2). In addition, hybrid-polytypes were designed by considering different stacking of two polymorphs. Herein, three hybrid-polytypes of α/β, γ/β, and α/γ have been investigated (Figure S3). Rotated polytypes are considered including α↶α, β↶β, and γ↶γ with rotation angles of 90, 21.79, and 90° for the upper layer, respectively (Figure S4). The rotated unit cell is chosen based on minimum mismatch of the top (rotated) and bottom (unrotated) layers. In addition, less than 100 atoms in the unit cell were taken into consideration.

Furthermore, we have generated single and bilayer carbon-doped phosphorene structures considering a constraint, namely, that in phosphorene with a 50 percent concentration of carbon, carbon atoms are not placed next to each other. Note also that, in bilayer structures, it is possible to place the atoms in two positions, which are represented by *m*_1_ and *m*_2_ (see Figure S5). For the later doped models, AA, AB, and AC stacking with *m*_1_ and *m*_2_ arrangements has also been investigated.

To evaluate the bond strength of atoms and obtain a measure of the thermodynamical stability of structures, the cohesive energy of bare and carbon-doped structures is calculated using eqs 1 and 2, respectively.
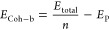
1
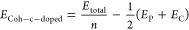
2where *E*_total_ and *n* are the total energy of the system and number of atoms in the system, and *E*_P_ and *E*_c_ the energy of isolated phosphorus and carbon atoms, respectively. The coefficient of 1/2 in eq 2 is due to the 1:1 carbon/phosphorus atom ratio in the structures.

### 2.1 Modeling the Field-Effect Transistor (FET) Configuration

DFT-based computations can be used for modeling field-effect transistors due to their ability to predict the properties of materials and interfaces at the atomic scale with rather good accuracy. Back in 2014, Brumme et al.^72^ designed a DFT-based approach to study doping effects in field-effect devices. Their method allows for calculation of the electronic structure as well as complete structural relaxation in a field-effect configuration. Here, we apply their approach to phosphorene-polytype-based field effect and analyze in detail the structural changes induced by the electric field. Following Brumme’s model,^72^ the phosphorene polytypes are placed in front of a charged plane or monopole—the location of the monopole in the unit cell is specified as “zgate” in the quantum espresso input—which models the gate (Figure 1b). The phosphorene polytypes layers are charged with an equal and opposite amount of *n*_dop_ × *A* charge where *n*_dop_ represents the number of doped electrons per unit area, and *A* stands for the area of the unit cell parallel to the surface. Consequently, a finite electric field is generated in the region between the gate and the system. To correctly determine the changes in the electronic structure and geometry for a given field-effect setup, the electric field within the vacuum region between periodically repeated images must be zero (the total potential should be constant). However, due to the dipole generated between the charged system and the gate, an artificial electric field is generated between the periodically repeated slabs. To overcome this effect, we introduce an electric dipole consisting of two planes of opposite charge located at the vacuum region adjacent to the monopole—in the quantum espresso input, the position of the dipole and the unit cell thickness are defined as “emaxpos” and “eopreg”, respectively. Following previous work,^70^ the optimal bipolar size is one-hundredth of the unit cell length, in line with the definition of dipole in the *z*-direction.

The application of an electric field between the gate and the system alters the atomic structure of the channel in the gate model, and it is necessary to perform a structural relaxation at the different fields of interest. Due to the opposing charges of the gate and the system during the structural relaxation, the atoms are displaced from their original positions and attracted to the gate. To prevent the movement of atoms toward the gate, a barrier potential is added between the system and the gate, which is equivalent to the dielectric structure placed between the channel and gate in the real FETs. Technically, the width and height of the potential barrier are defined in the quantum espresso input as “block_1”, “block_2”, and “block_height”, respectively. “block_1” and “block_2” represent the starting and ending points of the potential barrier, respectively, and the difference between these two determines the width of the barrier potential. The optimal size of the width barrier potential has been estimated as one-tenth of the unit cell length in the *z*-direction.^70,71^

Finally, the WanT code^73^ has been used to calculate electron transport and current–voltage curves. To investigate the transport properties of the candidate structures, we use the real space Green function method with the localized-orbital basis Hamiltonian constructed by using maximally localized Wannier functions (MLWFs). To this end, for each P and C atoms in the unit cell, we have positioned four sp^3^ and three sp^2^ hybrid orbitals, respectively, according to the definition of Wannier90.^74^ After wannierization, the spatial spread of each type of orbital is reduced to less than 10^–7^ Bohr ^2^. To show the accuracy of obtained MLWFs, the variation of the Hamiltonian with distance in the basis of Wannier function is shown in Figure S6 for the three desired structures in the absence of gate potential. Obviously, the band structure obtained from the Wannier functions completely matches the one build from Bloch functions. These confirm basis transformation has been carried out properly.

## 3 Results and Discussion

As already mentioned, polytypism applies to close-packed or layered materials, where polytypes are characterized by constituent layers with identical structures but different periodicities perpendicular to the layer plane (i.e., different stacking). Since layered materials have weak van der Waals (vdW) like interlayer interactions, polytypism is commonly observed and constitutes a highly relevant form of polymorphism for multilayer 2D materials.^75^ On the other hand, we recall that the main objective of the present work is to explore the FETs model of the phosphorene polymorphs which makes use of the flexibility and anisotropy of the phosphorene family in line with the need to use flat structures in the design of electronic devices.^76^ To this end, we make use of candidate structures in the gate model of the simple yet realistic FET model which is described in the previous section.

The beta phosphorene and phosphorene carbide structures are more stable than the other structures; their stability is in the range of −3.713, −5.483 eV per atom and −4.720, −5.176 eV per atom, respectively.

Interestingly, according to the literature, phosphorene carbide structures have the lightest electrons and holes effective mass^11,45^ leading to a high mobility of charge carriers in these structures.^77^ The stability and high mobility of charge carriers of phosphorene carbide suggest that this material may have good performance as a channel in the gated model, which can reveal details about the structural and electronic properties not easily accessible by experiments on FET. One of the key factors in the present FET model is the stability of the channel material in the different ranges of electric field because it is related to its FET performance as it is discussed in the following sections.

### 3.1 Geometric and Electronic Properties of Bare Phosphorene Polymorphs

As mentioned in the computational section, the studied structures include the α, β, and γ polymorphs (monolayer), homogeneous bilayers with different stacking, heterogeneous bilayers (α/β, α/γ, β/γ), and rotated bilayers (α↶α, β↶β, and γ↶γ). The unit cells of all monolayers and bilayers with three different stacking are shown schematically in Figures S1a, S2, S3, and S4.

Numerous theoretical and experimental data regarding structural and electronic properties for monolayer phosphorene, its allotropes and some of its bilayer structures have been reported.^78−80^ The present results of our systematic study on the three stable allotropes, namely, α, β, and γ, are summarized in Figure S1a and Table S1. A comparison with previous work shows good agreement between both sets of results^81−83^ and supports the use of these computational parameters in the calculations reported in the following subsections.

**Figure 2:**
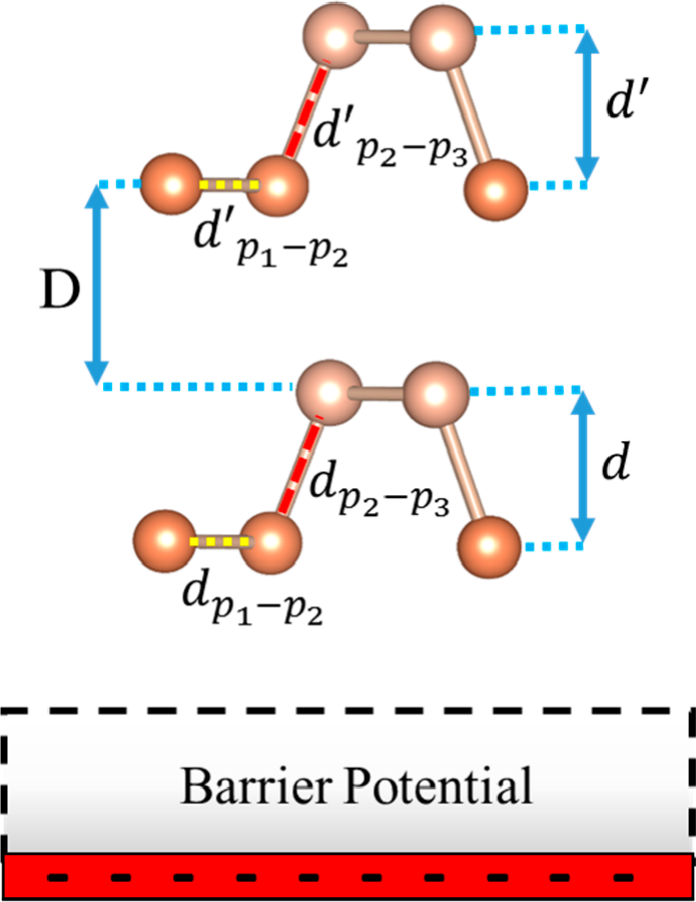
Schematic side view of the α phosphorene bilayer with the parameters defining the buckling layer in the *z*-direction, the distances between two top and bottom layers (*D*), and the different distances between atoms. Light and dark spheres represent the top and bottom atoms in each layer, respectively.

Phosphorene sheets have two types of P–P bonds in the relaxed structures. We marked the bonds parallel to the puckered layer as *d*_p_1_-p_2__ (bottom surface) and *d′*_p_1_-p_2__ (upper surface) as shown in Figure 2, while the bonds tilted to the two-dimensional in-plane directions are marked as *d*_p_2_-p_3__ (bottom surface) and *d′*_p_1_-p_3__ (upper surface), respectively, with the bond lengths for all structures reported in Table S2. In the monolayer structures, the largest *d*_p_1_-p_2__ (2.31 Å) and *d*_p_1_-p_3__ (2.26 Å) bond lengths belong to γ and β allotrope, respectively. In all homogeneous bilayer structures, the *d*_p_1_-p_2__ and *d*_p_2_-p_3__bond lengths in the top layer and *d*′_p_1_-p_2__ and *d′*_p_2_-p_3__ bonds lengths from the top and bottom layers coincide. The maximum and minimum of buckling height (2.11 and 1.24 Å; see Table S1) correspond to the α and β allotropes, respectively, while the γ allotrope exhibits an intermediate value of 1.49 Å. Among α, β and γ allotropes, β is the most stable one. According to the results reported for phosphorene,^57^ all bilayers are more stable than their constituent monolayers. This is also the case for heterogeneous structures.

For all stacking possibilities of homogeneous bilayers of α and β allotropes, the bonds parallel to the puckered layer remain the same as in the monolayer structure. But in the AA stacking of the homogeneous γ bilayer, there are noticeable changes with respect to the γ monolayer allotrope; −0.06, −0.01 Å in *d*_p_1_-p_2__ for the AA stacking and 0.03 Å in *d*_p_2_-p_3__ in the AC one. A noteworthy point in the obtained data for homogeneous rotated bilayer structures, where the top layer is rotated at a certain angle with respect to the bottom layer (α↶α (90°); β↶β (21.79°); γ↶γ (90°)) is that *d*_p_1_-p_2__ and *d′*_p_1_-p_2__ as well as *d*_p_2_-p_3__ and *d′*_p_2_-p_3__ increased. The details of the changes for three rotated structures are respectively as follows: α↶α: 0.45% (0.45%) and 0.00% (−0.44%), d (4.26%). β↶β: 0.44% (0.44%) and 0.44% (0.88%), *d* (0.00%). γ↶γ: 3.46% (2.16%) and 1.77% (0.88%), *d* (26.17%). These are compared to those of the monolayer structures (Table S2, Figure S7).

In the α/β heterogeneous bilayer structure, the mutual interaction of the two layers affects the bond length in the beta layer while the structure of the α layer remains unchanged. The mutual interaction effect in the α/γ heterogeneous bilayer structure causes changes in the bond length of the two constituent layers. Besides, in α/β and β/γ heterogeneous bilayers, the structure of the β allotrope becomes altered. Indeed, among the investigated allotropes, β is not only the most stable one but also induces the largest effect in heterogeneous bilayer structures. In all homogeneous bilayer stacking that involves the β allotrope, the buckling height of the individual layers remains almost the same as in the β allotrope. This is not the case for stacking involving α- and γ-allotropes, where a change in the buckling height is observed. In any case, in the homogeneous bilayer structures, the change is small and does not show a specific trend. It is noteworthy that the buckling height in the rotated bilayers is slightly changed compared with the constituent monolayers. In the heterogeneous bilayer structures, mutual interactions of constituent monolayers cause a change in the buckling height of both, top and bottom, layers. Distortion of buckling height of the α layer in the α/β and α/γ is 4.7% and 9.9%, respectively. Likewise, in the other layers of heterogeneous bilayers, the buckling height of constituent β and γ layers changes by 0.8% and 2.0%, respectively. Furthermore, for the β/γ heterogeneous bilayer, the buckling height of the γ layer remains unaltered, while the buckling height of the β layer experiences a mere 1.6% change compared to that of the monolayer structure.

The present results indicate that α^AB^, β^AA^, and γ^AA^ stacking exhibit the largest cohesive energy of −3.737, −3.746, and −3.897 eV per atom, respectively, (Table S2), in agreement with values in the literature.^50,84^ The largest cohesive energy for the γ^AA^ homogeneous bilayer is consistent with the fact that the γ monolayer is the less stable one. It is noteworthy that all rotated bilayer structures α↶α, β↶β, and γ↶γ show remarkable stability along with more (∼46%) (Table S2) cohesive energy as compared to the most stable unrotated counterparts. Another intriguing observation is the enhanced stability of α↶α compared to the other two rotated bilayers (β↶β and γ↶γ), even though its constituent allotropes are not individually the most stable ones. As previously mentioned, the heterogeneous α/β, α/γ, and β/γ bilayers exhibit higher stability than both the constituent monolayers and the homogeneous bilayers. The observed order of stability for heterogeneous bilayers is as follows: α/β> α/γ > β/γ. Although the cohesive energy for α and β monolayer is larger than that of the γ monolayer allotrope, the cohesive energy of α/β is larger than that of α/γ and β/γ with the values of 1.95% and 2.3%, respectively. As a result, the allotropes containing γ allotrope are less stable. Another valuable point is that, in general, heterogeneous bilayers are about 4% (Table S2) more stable than the homogeneous bilayers (Figure S7).

At the utilized computational level, α, β, and γ monolayers exhibit band gap values of 0.91, 1.9, and 0.43 eV, respectively. Analysis and comparison of band structures for homogeneous, heterogeneous, and rotated bilayers of phosphorene allotropes show that by adding a layer, the band gap decreases. Essentially, it has been reported that the interlayer stacking pattern plays a crucial role in determining the gap values.^47^ The present results (Figure S7) show that the largest band gap change occurs in all stacking structures involving the less stable allotrope (γ), which has zero gap. The band gap of structures involving the α allotrope is altered by at least 50% and at most ∼78%. The minimum variation of band gaps corresponds to the different stacking of the β allotrope, which is placed in the range of 12.6%. to 22%. The band gap is also reduced in the studied heterogeneous bilayers. As a conclusion of this part, in addition to the effect of increasing the number of layers on the band gap, a clear dependency appears of the band gap variation on the intrinsic properties of the constituent layers of the heterogeneous bilayer structures. For example, in the α/β and γ/β heterogeneous bilayers, sharing the β layer, the second layer is composed of other allotropes (α and γ), the band gap decreases from 1.66 eV (homogeneous bilayer (β^AA^)) to 0.52 eV (α/β) and 0.74 eV (γ/β). This means that creating multiple layers of different allotropes can be a means of controlling the band gap (Table S2 and Figure S7).

### 3.2 Structural and Electronic Properties of Bare Phosphorene Carbide

In recent decades, numerous stable two-dimensional allotropes of monolayer PC were theoretically predicted.^45,85^ Afterward, few-layer two-dimensional PC was synthesized successfully via a novel carbon doping technique.^86^ In this section, we present findings concerning the stability and electronic structure of PC models for the α, β, and γ phosphorene polymorphs as well as the homogeneous bilayers (Figures S8–S11). Related structural details and binding energies are summarized in Table S3.

The structural optimization of α, β, and γ phosphorene layers doped with C in a one-to-one ratio results in a single shared structure hereafter referred to as CPCS (common phosphorene carbide structure) and displayed in Figure S8. This structure has a configuration similar to that of the bare β allotrope. Moreover, similar to those of other phosphorus carbide monolayers,^44,45^ CPCS has zero band gap. It is 29.38%, 28.96% and 33.27% more stable than the undoped α, β, and γ structures, respectively (Tables S2 and S3). The bonds lengths and angles obtained for CPCS monolayer are in good agreement with the reported values^45,52,53^ of carbon-doped phosphorene structures.

Although the carbon-doped α, β, and γ monolayer structures were all optimized to a CPCS, the doping of homogeneous bilayer structures of α, β, and γ with carbon according to the conditions that we mentioned earlier leads to distinct structures for each of the allotropes. In the homogeneous bilayer of αc structures, including α_PC-m1_^AA^, α_PC-m2_^AA^, α_PC-m1_^AC^, α_PC-m2_^AC^, and α_PC-m2_^AB^, a new interaction with covalent bond characteristics appears (see Figure S9). In the α_PC-m1_^AA^ structure, the mentioned covalent interaction involves carbon and phosphorus atoms from two constituent layers, resulting in the formation of six-member rings. However, in the α_PC-m2_^AA^, α_PC-m1_^AC^ and α_PC-m2_^AB^structures, a new strong interaction forms between two phosphorus atoms of the constituent layers. This leads to the formation of similar ten-member rings in the α_PC-m2_^AA^ and α_PC-m1_^AC^ structures, while in the α_PC-m2_^AB^structure, four-membered rings are formed (see Figure S9).

Except for β_PC-m2_^AC^, in the homogeneous bilayer of the βc structures, the situation is different. In these structures, due to the high interlayer distance, no covalent bond is formed between the two constituent layers. In the β_PC-m2_^AC^ structure, due to the arrangement of the atoms, a covalent bond is formed between P and C atoms from top and bottom layers, which led to 6-member rings in these structures (see Figure S10).

The characteristic feature of γc homogeneous bilayer structures is the formation of four, six, eight, and ten-member rings (Figure S11). Covalent bond forms between the two constituent layers in all structures of these doped allotropes. In the γ_PC-m1_^AA^, γ_PC-m1_^AC^and, structures, the rings are made up of six atoms. It should be noted that the optimization of γ_PC-m1_^AA^and γ_PC-m1_^AC^ structures leads to a shared single structure. The strong interactions between C and P atoms, enabled by the sliding of vertically stacked layers, facilitate the formation of eight-membered and four-membered rings in the structure γ_PC-m1_^AA^ and γ_PC-m1_^A^ structures. In theγ_PC-m1_^AB^ structure, the covalent bond formed between the carbon and phosphorus atoms from constituent layers leading to the formation of six-member rings in these structures. Typically, the covalent bond length between the two constituent layers falls within the 1.83–2.22 Å range. All structural parameters related to the examined structures are reported in Table S3.

### 3.3 Properties of Gated Monolayer and Polytypes

To assess the suitability of the phosphorene monolayer and polytypes for application in field-effect transistor channels, the monolayers and bilayers were placed at the center of the region as in Figure 1, with a constant external electric field. To minimize interactions between bilayers in neighboring supercells, a large supercell size of 30 Å was chosen. The arrangement of structures in the gate model follows a similar grouping as in the geometric and electronic studies section. Since the change in the structural parameters affects the electronic properties, electron transport and performance of the structures as FET channel, we first examine the electronic structure details in the presence of the gate and subsequently discuss the electronic properties including the details of the band structure, charge density distribution and electron transport. To this end, single and bilayer systems with different doping hole levels ranging from 0.05 to 2 holes per unit cell with increment of 0.5 were selected. Hole doping was considered because, as mentioned in the computational part, the potential of the gate is equal to the charge of the system with the opposite sign. Furthermore, we recall that in field-effect transistors with p-type channels, the gate potential must be negative, otherwise the transistor will not perform properly.^87^ The criterion for selecting this range for the hole loading to the system was the stability of the system versus the loading amount. As a measure of the stability of the systems, the resistance of phosphorene to tension was chosen. According to a report by Wei et al.,^88^ α-phosphorene polytype exhibits a tensile strength of 30% in the armchair direction. Further application of tension can result in a structural change. Subsequently, we compared the collected data with the results obtained after applying potential gates up to 2 holes per unit cell. The findings revealed that hole doping exceeding 1.5 holes per unit cell on α-phosphorene led to a decrease in the cohesive energy compared to the state in which a 30% tension was applied to it. Hereupon, we have limited the amount of doping up to 2 holes per unit cell. We have included data for doping 2 holes per particle to show the instability of the structures and its effect of the different properties. It is worth mentioning that the degree of effectiveness of the systems by the created electrical field between the system and gate depends on the potential value of the gate as well as the distance of the system from the gate.

### 3.4 Atomic Structures within the Channel of Field-Effect Transistors

Since, for homogeneous bilayer polytypes with different stackings (α^AA^, α^AB^, α^AC^, β^AA^, β^AB^, β^AC^, γ^AA^ γ^AB^, and γ^AC^), the geometric changes for the monolayer closer to the gate in the optimized bilayer structures are the same as the changes of the bare monolayer of allotropes in the gated model, we present only the results of optimization for the homogeneous bilayers of α^AA^, β^AA^ and γ^AA^. We recall that the increase of the layer number causes to stability of the structures.

In all homogeneous bilayer structures, van der Waals interaction between the layers led to the increase of the top layer distance from the gate. Moreover, bilayer structures contain a higher number of atoms within the same surface area compared with their monolayer counterparts. Consequently, the top layer in all bilayer structures is less affected by the gate potential than the bottom layer (closer to the gate). The results show that the general arrangement of atoms in bilayer structures of homogeneous β^AA^, β^AB^, β^AC^, γ^AA^ γ^AB^, and γ^AC^ remains the same as their monolayer structures under the gate potential and there is no change by increasing the potential. But in the α^AA^and α^AB^ bilayer structures, increasing the gate potential changes the structure of the layer closer to the gate, as for the bare phosphorene monolayer, and its thickness increases. In the presence of a gate potential, the α^AC^ stacking bilayer has enhanced stability when compared to α^AA^ and α^AB^ stacking. In the α^AA^ structure, the interlayer distance decreases as the gate potential increases, so that at the potential of 2 holes per unit cell, the interlayer distance decreases by ∼9%, compared to that of the bare structure. The distance decrease between layers causes the upper layer to be closer to the gate, the closer to the gate the more affected by the gate potential. At a 2 hole per unit cell potential, the thickness of the mentioned monolayer (*d*′) increases by 1.10%. The effect of the potential on the layer closer to the gate is an ∼34% increase in the thickness of this monolayer (*d*) at a potential of 2 holes per unit cell compared to a bare counterpart. In the α^AB^ and α^AC^ cases, with the continued increase in the gate potential, the interlayer distance begins to expand. Specifically, at a potential of 2 holes per unit cell, there is an increase in the distance between the layers within the structures α^AB^ andα^AC^ by ∼14.18% and ∼13%, respectively, compared to that of the bare structures. In both α^AB^ and α^AC^structures, the thickness of the layer (*d*′) far from the gate remains unchanged by applying the gate potential, but in the layer closer to the gate, as in the α^AA^ bilayer, the thickness of the monolayer (*d*) increases by increasing the gate potential (Figure S12). It is worth noting that the α^AC^ structure exhibits a lower increase in monolayer thickness (*d*) compared to α^AA^and α^AB^. This distinction in the trend of variation of structural parameters is the only difference observed. In the β^AA^and β^AB^ bilayers, the interlayer distance (D) decreases by increasing the gate potential, while in the β^AC^structure, it increases by ∼30%, compared to that of the bare state. Bilayers of β^AA^, β^AB^, and β^AC^ are different from homogeneous alpha phosphorene bilayers; as the gate potential increases, the thickness of the layer closer to the gate decreases with a trend similar to that of the β monolayer. Also, by applying the gate potential, bond lengths in these three structures decrease. In all studied stackings of the γ bilayer, all bond lengths in the layer closer to the gate decrease and those of in layer further away the gate increase.

In heterogeneous bilayer structures, the bond lengths and bond angles experience slight alterations while the overall atomic arrangement of the structures. However, by increasing the gate potential up to 2 holes per unit cell in the β/α structure, the thickness of the β layer (*d*) decreases by 4.16% and that of the α layer increases by 1.33% compared to those of the bare states. In the γ/α structure, the thickness of the γ (*d*′) decreases by 0.66% and that of in α increases by 2.10% and in the structure β/γ thickness of β decreases by 4.20% and that in γ increases by 0.66%. The layers’ distance in β/α, γ/α, and β/γ decreases by 2.48%, increases by 0.6%, and decreases by 3.48%, respectively.

The obtained results from the relaxation of the atoms in the primary unit cell of α↶α show that the in-plane bond length is increased by 0.44% in both layers compared to that of the phosphorene monolayer. The thickness of phosphorene monolayers (*d*) in this structure has increased by ∼4% compared to that of the phosphorene monolayer. This increase is attributed to the angle changes observed in this structure compared to those in the phosphorene monolayer.

The phosphorene monolayer has anisotropy^89−91^ and this feature plays an important role in the properties of the rotated homogeneous bilayers and results in different characteristics from the homogeneous bilayers. For this reason, the structural changes caused by applying the gate potential are less in the rotated homogeneous alpha–alpha bilayer compared to the homogeneous bilayer and monolayer of phosphorene. In the 90° rotated bilayer γ↶γ, the thickness of each monolayer increases by 20.74% and 25.78% for the bottom top layer, respectively, compared to that of monolayer γ-phosphorene. The thickness of the bottom and top layer as well as the distance between the two layers in the β↶β structure at the gate potential of 2 holes per unit cell decreases by 3.33%, 3.07%, and 2.22% compared to those of the bare structures, respectively. In rotated structures, the overall arrangement of the structures does not change by applying the gate potential.

**Figure 3:**
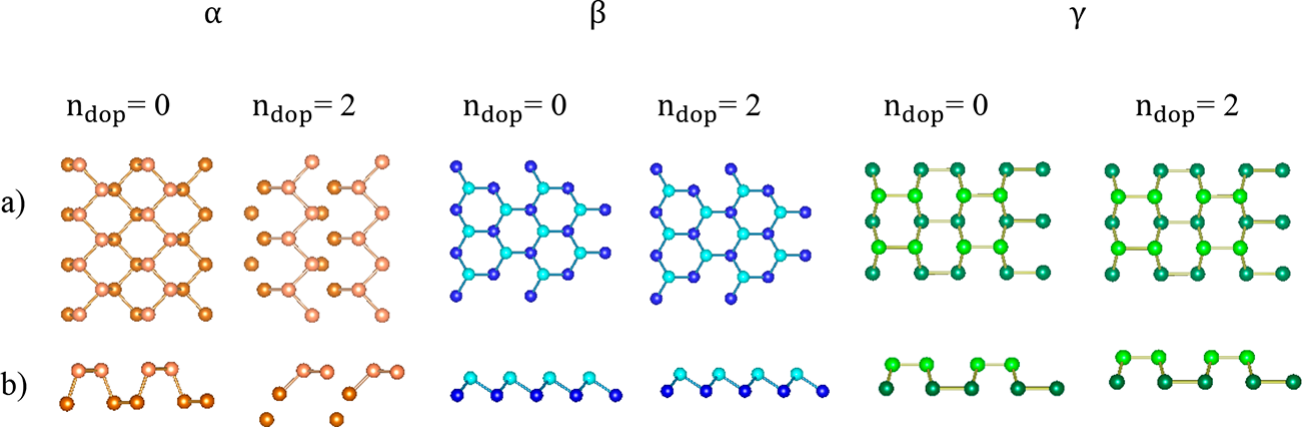
Structures of α, β, and γ phosphorene from the top and side views in the absence of the gate potential (n_dop_= 0) and in the presence of a gate potential (*n*_dop_= 2 hole per unit cell).

Summing up, while all three phosphorene allotropes, α, β, and γ, possess an identical number of electrons and atoms in the primary unit cell, they display varying structures as well as distinct electron density distributions within these structures. (Figure S1). Therefore, different trends and ratios of changes in the bond lengths and angles were found in these structures under application of the gate potential. Upon closer examination of the data, it becomes apparent that in α and γ-phosphorene structures, three types of bonds exist that exhibit varying degrees of effectiveness in response to the gate potential. This is because of the different positions of atoms and their structure. In α-phosphorene, the longer out-of-plane P–P (*d*_p_1_-p_2__) bond length than the in-plane (*d*_p_1_-p_2__) bond length causes easier bond breaking by increasing the gate potential. Moreover, the atom closer to the gate moves toward the gate and leads to the increment of roughly 35% in thickness compared to that of the bare state and hence an overall change in α-phosphorene structure (Figure 3). In contrast to the α-phosphorene structure, the overall arrangement in γ-phosphorene remains unchanged even when a gate potential is applied. This is attributed to the nonplanarity of the γ-phosphorene structure in the *z*-direction (Figure 3). In the β-phosphorene structure, similar to the results found for α- and γ-phosphorene structures, the positions of the atoms differ from each other. However, due to the buckled structure and high symmetry of β-phosphorene, all three bonds occupy the same position relative to the gate potential, resulting in equal changes in bond lengths. Additionally, the increase in angles of β-phosphorene caused by the application of the gate potential leads to a decrease in thickness.

In the CPCS structure, with an increase in the gate potential, the closer in-plane phosphorus–carbon bond length to the gate potential decreased. The thickness (*d*) of the CPCS structure decreases by increasing the gate potential and reaches its minimum value at the gate potential of 1.5 holes per unit cell (151.22% decrease compared to that of the bare state). At the gate potential of 2 holes per unit cell, the thickness increases compared to 1.5 holes per unit cell, but at the end, the thickness of the CPCS structure at the gate potential of 2 holes per unit cell is still lower compared to that of the bare state.

The results in this subsection demonstrate that the gate potential causes formation of a quasi-flat structure and results in a CPCS which has similar structure to graphene. A comparison of the structural data for undoped and doped counterparts shows that the effectiveness of doped bilayers by the gate potential is larger than in the undoped structures, in such a way that by applying the gate potential, to most of the doped bilayers their overall structures are changed. This indicates that the range of gate potential for no structural change is lower in the doped structures compared to undoped ones.

The overlap between phosphorus and carbon atoms in the doped structures led to the formation of modified structures. However, it is important to note that this overlap is not as big (size and difference in energy levels) as the overlap between phosphorus atoms in phosphorene or carbon atoms in graphene. This heightened sensitivity leads to substantial structural modifications. These changes are so significant that they trigger a phase transition within the material. This characteristic proves to be advantageous for these structures, particularly in the presence of an applied potential.

To close this subsection, note that the cohesive energy results show that, after applying the gate potential, the stability of the structures tends to decrease. The order of the highest reduction percentage of cohesive energy in α and its bilayers is as follows: α > α^AB^ > α^AA^ > α^AC^. The same trend was found for β and γ allotropes except that the structural stability reduction percentage is lower than that of α-phosphorene. In the carbon-doped structures, the application of gate potential reduces the cohesive energy. It is noteworthy that by applying the maximum gate potential (in our work), the cohesive energy of the carbon-doped monolayer structure is greater than that of the initial α-phosphorene while maintaining the overall arrangement.

### 3.5 Electronic Structure within the Channel of Field-Effect Transistors

Previous experimental and theoretical studies on graphene and MoS_2_ have shown that it is possible to tune the band gap by applying an external electric field.^92−97^ Therefore, it is interesting to know how the band gaps of the phosphorene family introduced in this study change with application of the gate potential.

Apart from the alterations in electronic structure induced by the gate potential, the results obtained indicate that even the smallest gate potential applied to the studied structures can cause a phase transition from a semiconductor to a conductor. Conversely, structures that are inherently conductive maintain their phase and retain their conductivity when the gate potential is applied. With the exception of the CPCS structure and of γ_PC-m_1__^AB^, γ_PC-m_2__^AC^, α_PC-m_2__^A**B**^, and α_PC-m_1__^AA^ bilayer structures, which undergo two phase changes in their band structure with varying gate potential, other phosphorene polymorphs transition occurs from being semiconductors to conductors.

As the gate potential increases, the valence bands in these structures shift above the Fermi level. Moreover, as the gate potential continues to increase, the displacement of the valence band also increases.

The specifics of the changes in the band structures of the studied structures are outlined below.

From the band structure of α and α^AA^ at different gate potentials, it can be concluded that at a 2 hole per unit cell potential, the band structure qualitatively changes compared to small changes at lower potentials. In monolayer, homogeneous bilayers, and rotated bilayers of beta phosphorene, like alpha phosphorene, the semiconductor phase changes to metallic phase by applying the gate potential. Bilayers of gamma phosphorene, unlike alpha and beta structures and their bilayers, retain their phase in the presence of the gate potential and the phase remain metallic. As mentioned, the CPCS structure is intrinsically conductive and it remains as so at gate potentials up to 1.5 hole per unit cell. However, at a potential of 2 holes per unit cell, the electronic structure changes from conductor to a semiconductor with an indirect band gap of 2.15 eV (Figure S13). In the bilayers of alpha phosphorene carbide, α_PC-m_1__^AA^, and α_PC-m_2__^A**B**^, which are conductive in the absence of a gate potential, the conductive character remains by applying a gate potential up to 1.5 hole per unit cell, but at a potential of 2 hole per unit cell their character changes from conductor to semiconductor, respectively, with a direct band gap of 1.52 and 1.17 eV. In the β-phosphorene carbide bilayer structures, the application of the gate potential causes the band gap to close, and it remains metallic in the whole range of applied potentials. In the bilayer structures of gamma phosphorene carbide, γ_PC-m_1__^AB^, and γ_PC-m_2__^AC^ structures, which are intrinsically semiconducting, become conductive under the application of a gate potential. However, by further increasing the gate potential up to 2 holes per unit cell they change from conductors to semiconductors with indirect band gaps of 1.14 and 0.68 eV, respectively.

To further investigate the effect of the gate potential in the electronic structure of phosphorene polymorphs, we analyze the electron density distribution, which is one of the characteristics of any material. In addition, by considering structure phosphorene as a suitable reference,^98^ the electron density difference (Figure S14), and chemical intuition, are proper tools to extract the structural changes.

**Figure 4:**
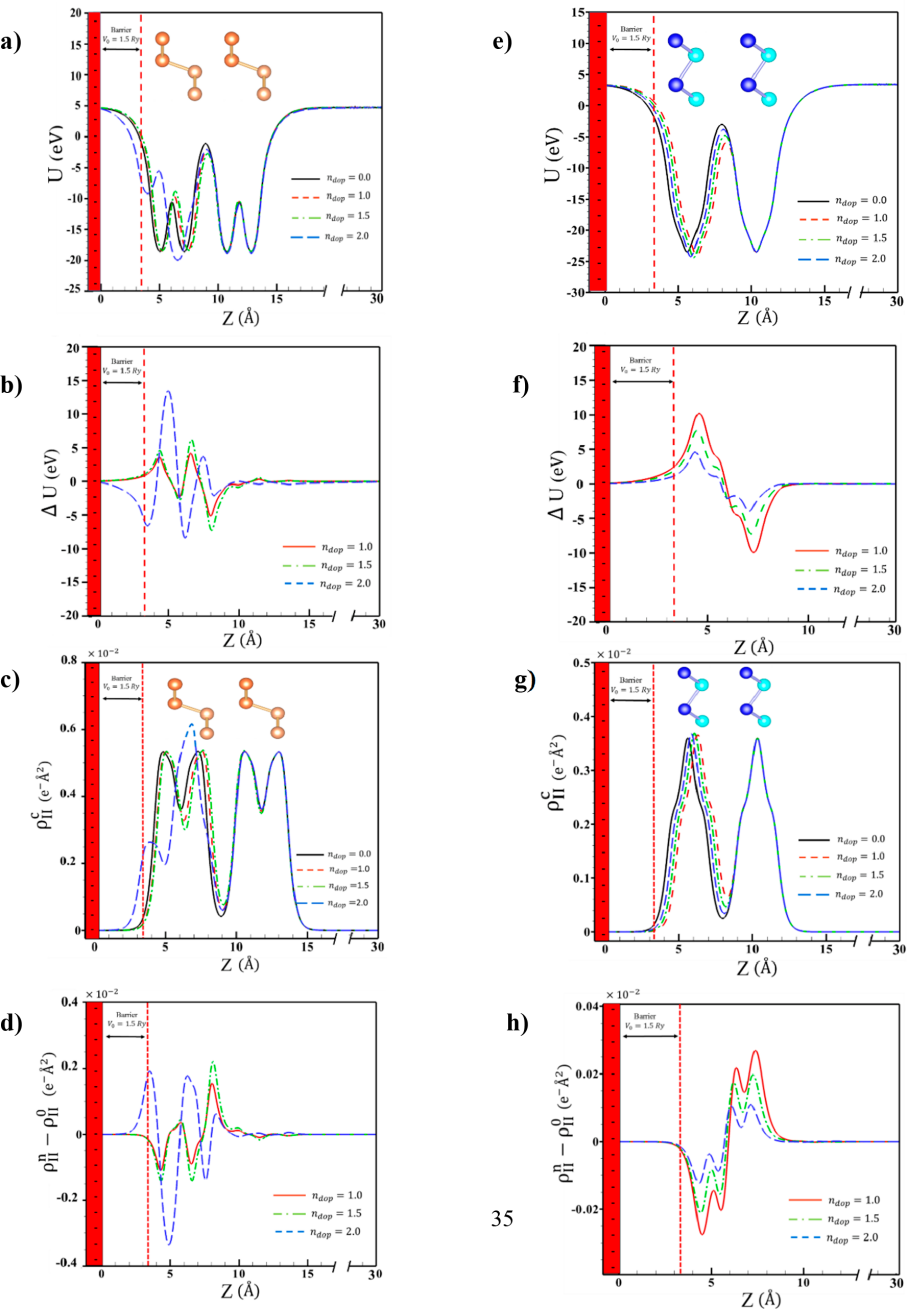
Averaged potential (a, e), averaged charge density (c, g), difference between the potential (b, f) and difference between the charge density (d, h) of the doped and undoped α^AA^ and β^AA^ bilayers as a function of the *z*-direction.

Due to the physical arrangement of P atoms and the quasi-two-dimensional structure of these materials, the electron density of α, β, and γ phosphorene monolayers have two, one, and one peak(s), respectively. The so-called phosphorene polymorphs have two half-plane structures. The double-peaked electron density in α indicates that the density of electrons between these two half-planes is lower than inside the half-planes, which is a reason for the *d*_p_2_-p_3__ being longer than *d*_p_1_-p_2__ (in-plane bond). The single peak of the electron density diagram of β and γ phosphorene indicates that, unlike in the α one, the accumulation of electrons between the two half-planes is larger than in the half-planes inside, and this is a reason for the shorter *d*_p_2_-p_3__ distance compared to that of d_p_1_-p_2__ in the mentioned structures. The results obtained from the calculation of the electron density and potential energy at different gate potentials show that the gate potential changes the density distribution of electrons in different structures, Figure 4.

Although there is a correlation between the changes in different regions of the α studied structures and different parts having a mutual effect on each other, this change is larger in the layers and atoms closer to the gate than in the layer and atoms further away from the gate. To better show the changes trend of electron density and potential energy, the variation of these features is shown in Figure 4 for the α and β allotropes which belong to the most unstable and stable structures in the presence of the gate potential, respectively. As shown in Figure S14, changes in potential energy and electron density in the β^AA^ structure are insignificant, and the arrangement of atoms remains similar to that of bare AA staking of the β allotrope. However, in the α^AA^struture, in the layer closer to the gate, potential and electron density changes are noticeable, which causes a change in the arrangement of atoms and, as a result, induces changes in the properties of the related structures.

Figure 4 compares the potential energy and electron density at different gate potentials for the isolated structures. The disparity in potential energy and electron density increases with increasing gate potential. In the α allotrope, the difference from the isolated structure is much larger in the layer closer to the gate. The later trend has also been found for the β allotrope but the magnitude of changes in both layers is the same and symmetrical.

In bilayer α phosphorene structures, the trend of changes in structural and electronic properties induced by the presence of the gate potential is similar to that observed in the phosphorene monolayer, but compared to bilayers of other allotropes, the changes in electron density and potential energy in the α bilayer induced by the presence of the gate potential are larger. In addition, in the gated bilayer structures of all studied allotropes, increasing the gate potential causes an increase in the electron density between the two layers, and as a result, an increase in the potential between the two layers of AA and AB stackings. However, in the AC staking of all three allotropes, the electron density between the two layers decreases when the gate potential. It is therefore clear that the layers close to the gate are more sensitive to the presence of the applied potential than the layers far from the gate. Nevertheless, understanding the electronic and structural changes in the bilayer induced by the presence of the gate sheds light on several complications in estimating the behavior of the layer in the presence of the gate potential. For example, the comparison of the α^AA^ and β^AA^ structures in the presence of the gate potential shows that in the β^AA^ structure, unlike the α^AA^ structure, the layer farthest from the gate potential is not affected by the gate potential. While there is effectiveness in the farthest α^AA^ layer and the bond length, bond angle and layer thickness change by 0.88%, 0.35% and 1.1%, compared to the values for the bare structure (Figure S14 and Table S3).

Another noteworthy point is the symmetric or asymmetric effectiveness of the gate potential for the nearer layer. As can be seen in the Figure S12, in the layer close to the gate potential, the angles and bond lengths in the β^AA^ structure are symmetrically affected by the gate potential. In such a way that symmetrical changes, in the direction of an increase in one section and a decrease in the other part, in different parts of the layer close to the gate eliminate the effect of the gate potential on the structural deformation. In other words, the resulting average of the variations due to gate potential does not show a change in the structure, and the overall arrangement of the structure is preserved. On the other hand, in the α^AA^ structure, the atoms and thus the bond angles and lengths are symmetrically unaffected by the gate potential. For this reason, the effect of the potential in different parts of the layer near the gate is different, and this has caused a general change in the structure.

As stated in the structural properties section, doping phosphorene bilayer allotropes with 50% carbon atom does not change the stability trends of bare bilayer allotropes. In this regard, in α phosphorene carbide structures, the effect of the gate potential on the electron density and potential energy of these structures is as high as in the undoped α phosphorene bilayers. Consequently, in some of these structures, the electron density and potential energy change significantly. Analysis of the electron density and potential energy of β phosphorene carbide structures shows that the gate potential effect in AB and AC stackings is such that the electron density and, as a result, the interaction between the two layers decreases and the distance between the two layers increases. In the AA arrangement of this group, the gate potential causes a slight change in the arrangement of atoms closer to the gate, increases the electron density and interaction between the layers, and decreases the distance between the layers. Except for γ_PC-m_2__^AA^ and γ_PC-m_2__^A**B**^ structures, the changes of electron density and potential energy of γ phosphorene carbide structures are the same as for the β phosphorene carbide. In the two γ_PC-m_2__^AA^ and γ_PC-m_2__^A**B**^ structures, the electron density in the layer farthest from the gate does not change, although in the layer of both mentioned structures closer to the gate, there are significant changes leading to an overall structural change.

We can summarize and explain the shift of energy levels and changes in the band widths based on structural alterations and their impact on the electron density distribution. Modifications to the structural characteristics of materials can have a profound impact on their electronic properties. In the specific case of the phosphorus family, these alterations can significantly influence the distribution of electron density within these structures. On the other hand, electron density distribution plays a crucial role in determining various electronic properties, such as conductivity and resistivity. When the distances, angles, buckling, and overall structure of a material like the phosphorene family are modified, they can directly affect how electrons are distributed within the material. By understanding the changes in electron density distribution resulting from structural modifications, we can gain a deeper understanding of the material’s electronic properties (Figure S14). The direct impact of these changes in the structures is observed in the electronic band structure, including variations in bandwidth and band shifts.^99^ Consequently, in the mentioned structures, the valence band has shifted toward positive energies.

According to this part, it is interesting to notice that the CPCS structure displays case desirable attributes such as a phase transition from a conductor to a semiconductor, enhanced stability compared to other studied allotropes through carbon atom doping, reduced buckling in the structure upon applying a gate potential, and the obtained graphene-like structure with favorable properties^100−102^ in the presence and absence of the gate potential.

### 3.6 Quantum Transport

The quantum description of the electronic conductance is a complex nonequilibrium problem, and electric transport and FET characteristic of the pristine and C-doped phosphorene’s should be investigated by nonequilibrium Green’s function (NEGF) method. Hence, the present work constitutes the first step in focusing on structural and electronic properties. Thus, real space Green’s function formalism based on the Landauer approach is taken into consideration for the initial assessment. Accordingly, the electron transport and electric current of the α (low stability) and β phosphorene (high stability) and of CPCS among doped structures with desirable attributes are examined and compared.

**Figure 5:**
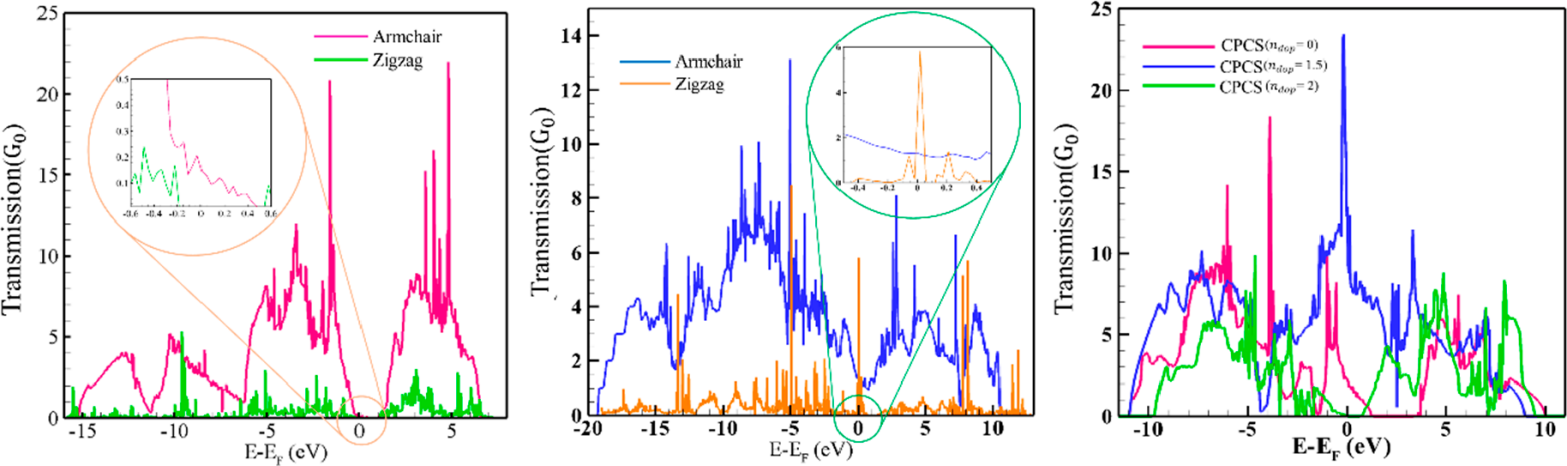
Comparison of the electron transport in armchair and zigzag directions for α (left), β (middle) phosphorene, and CPCS (right).

**Figure 6:**
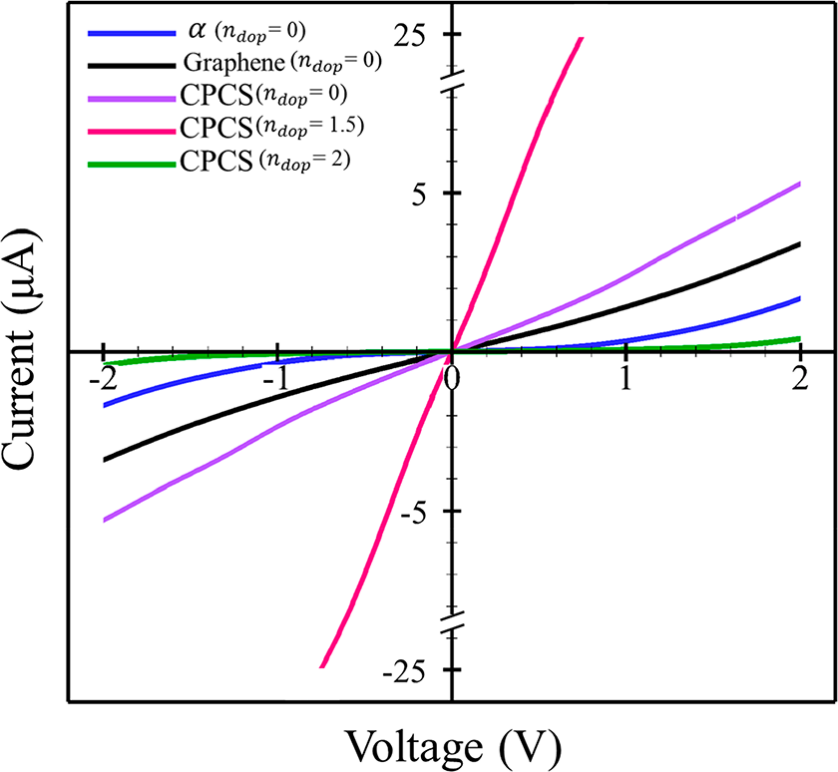
Current–voltage (*I*–*V*) curves for, graphene, α phosphorene, and pristine and charged CPCS at *n*_dop_= 1.5 and *n*_dop_= 2 hole per unit cell.

**Figure 7:**
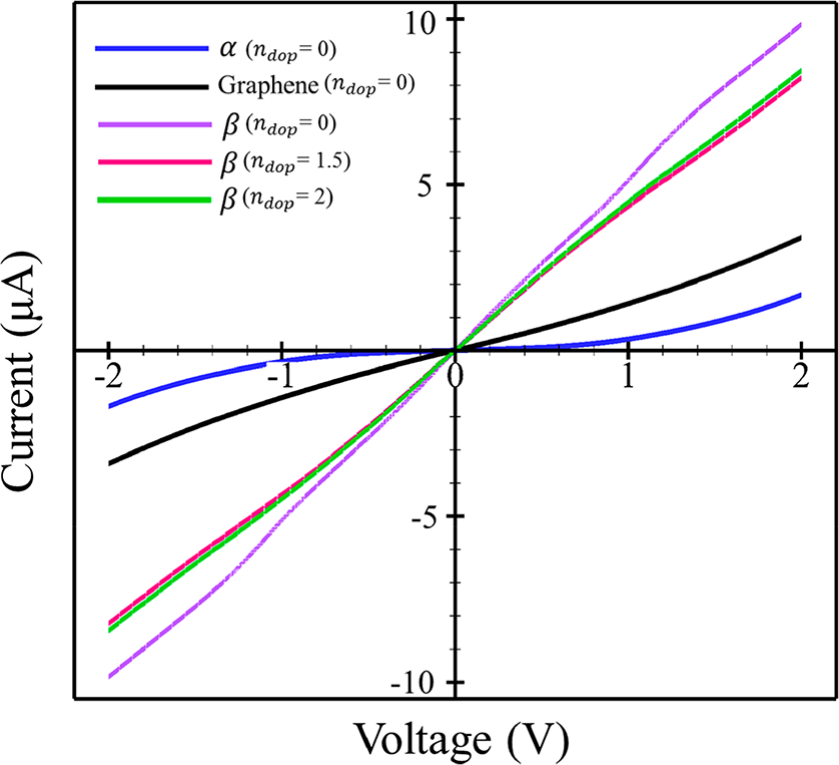
Current–voltage (*I*–*V*) curves for graphene and pristine and charged α and β phosphorene.

The results of electron transport calculations for the mentioned structures are summarized in Figure 5 and those for current–voltage in Figures 6 and 7. In the absence of gate potential, phosphorene does not exhibit electron transport and, consequently, electronic current either in both armchair or zigzag directions at the Fermi level.^83^

The CPCS has electron transport at gate potentials of 0 and 1.5 holes per unit cell (Figure 5c) which is due to the density of the electronic states in the Fermi level of this structure. This structure has the highest amount of electron transport and electronic current at a potential of 1.5 hole per unit cell in both directions in comparison with graphene. The calculated CPCS band structure (Figure S13) shows a band gap at potential equal to 2 holes per unit cell, which implies the absence of available density of electronic states with concomitant zero electron transport and electronic current at the later potential.

In the absence of gate potential, the composition and arrangement of C and P atoms in the CPCS structure lead to a higher passing current through this structure than in graphene. The comparison of the band structure and Fermi energies indicates that in the CPCS structure, there is no band gap, and the Fermi energy is lower compared to graphene. The reason for these changes can be attributed to the charge transfer from phosphorus atoms to carbon atoms (∼0.1) and the inherent potential of carbon atoms in this structure to accept charge. This results in reduced charge accumulation on phosphorus atoms, leading to decreased repulsion, increased overlap, and consequently flattening of the structure. In other words, the structure under consideration exhibits behavior similar to graphene, except for the higher value of electron density per unit surface area.

Applying an electric field increases the current passing through the CPCS structure in such a way that in an electric field equal to 1.5 holes per unit cell, the electric current reaches its highest value compared to current values in other gate potentials. The higher current observed in CPCS structures compared with graphene can be attributed to the value of electron density per unit surface area. This higher electron density in CPCS structures translates to a more efficient flow of the electric current through the material. Due to the fact that the application of an electric field equivalent to 2 holes per unit cell changes the phase of this structure from conductor to semiconductor with a band gap of 2.15 eV, the current passing through the CPCS structure in this field (2 holes per unit cell) is largely reduced. Consequently, by changing the electric field, it is possible to control the current passing through CPCS-based devices in different conditions.

According to the present I–V curves (Figures 6 and 7) and in agreement with previous studies,^84,103^ the current passing through graphene is high. But in the β phosphorene structure (Figure 5 and Figure 7), the electron transmission and passing current is larger than that predicted for graphene and phosphorene. Although gated β phosphorene has a reduced current through this surface in higher gate potential, the current is still higher than that of graphene. Increasing the electric field does not have a great effect on the current value in this structure.

## 4 Conclusions

Understanding the principles of 2D field-effect transistors is important for solving practical manufacturing problems. It has been claimed that a deeper understanding of the discoveries of these transistors at the fundamental level will strengthen their market value.^104^ In the present work, a fresh and comprehensive picture of the phosphorene polytypes and their carbon-doped counterparts has been presented and their properties related to a possible use in FET have been discussed.

The α, β, and γ structures of phosphorene allotropes have the same number of electrons and atoms in the designed unit cell. However, due to their placement in different regions of the potential energy surface minima, they exhibit different structural and electronic properties with respect to each other. In this respect, the structural properties of the homogeneous bilayers and the rotated bilayer for each allotrope resemble the monolayer structure of each of them.

The doping of carbon atoms to the bilayer structures of phosphorene allotropes causes significant changes in the positions of atoms and the interlayer distance compared with the original bilayers. These fundamental changes lead to the formation of a new type of covalent bonding between the layers in several PC structures.

The monolayer and bilayer structures of β phosphorene have the highest band gap compared to those of other structures. Doping with carbon atoms in the structures leads to a fundamental change in the band structure due to the alteration of the structural properties. So in CPCS and bilayer structures, the band gap is closed and conductivity is induced in these structures.

Using the studied structures as channel materials in field-effect transistors causes the structural properties of the systems to change upon application of the gate potential. The extent of the structural property changes increases with an increase in the gate potential. The sensitivity of the monolayer and bilayer structures with different stacking arrangements of α phosphorene and several related structures is higher compared with other arrangements. The results of the structural stability analysis indicate that bilayer formation, including different stacking arrangements, layer mismatch, layer rotation relative to each other, and substitution of carbon atoms with a 1:1 ratio of phosphorene atoms in the structures, enhances their stability.

Furthermore, the present results obtained from periodic calculations using density functional based methods, predict that in the presence of an electric field of up to 2 hole per unit cell, the CPCS and β structures are more stable than the α and γ polymorphs in good agreement with the results reported by Zhen et al.^105^ Moreover, in the β and CPCS structures, the electron transport is also larger than that predicted for the other studied systems. Therefore, applying the gate potential induces an increase of the current passing through CPCS and it reaches its highest value, compared to other fields, at the gate potential of 1.5 holes per unit cell. Finally, a phase transition from conductor to semiconductor in the CPCS structure, induced by the presence of an electric field equivalent to 2 holes per unit cell, causes significant electric current reduction. Consequently, it is possible to tune and control the current passing through CPCS-based devices under different conditions by changing the electric field. The present work suggests that CPCS and β structures are promising 2D materials for channels in FET devices.

## Supporting Information Available

Structural information on all studied systems, relevant band structures and charge density plots (PDF)
