# Supplementary material for: Electrostatic Gating of Phosphorene Polymorphs
Source: J Phys Chem C Nanomater Interfaces. 2024 Feb 9;128(7):2997–3010. doi: 10.1021/acs.jpcc.3c05876 (PMC10895923; doi:10.1021/acs.jpcc.3c05876)
Supplement: Supplementary file 1 — jp3c05876_si_001.pdf [file jp3c05876_si_001.pdf]

Supporting Information

Electrostatic Gating of Phosphorene Polymorphs

Fereshteh Mahmoodpouri Malayee,[1] Robabeh Bagheri[1], Fariba Nazari,[1,2] Francesc Illas[3]

[1]*Department of Chemistry, Institute for Advanced Studies in Basic Sciences, Zanjan 45137-66731, Iran*

[2]*Center of Climate Change and Global Warming, Institute for Advanced Studies in Basic Sciences, Zanjan 45137-66731, Iran*

[3]*Departament de Ciència de Materials i Química Física & Institut de Química Teòrica i Computacional (IQTCUB), Universitat de Barcelona,C/Martí i Franquès 1, 08028 Barcelona, Spain*

Contents

Table S1. Crystal structure information of the most stable individual phosphorene allotropes.

Table S2. Structural parameters including cell parameters (a, b), Bond length ($d_{p1\text{-}p2,}$ $d_{p2\text{-}p3)}$, thickness of this single layer (d), interlayer distance (D) binding energy and band gap of bare α, β, γ, α^{AA}, α^{AB}, α^{AC}, β^{AA}, β^{AB}, β^{AC}, γ^{AA}, γ^{AB}, γ^{AC}, α/β,α/γ,β/γ and rotated bilayers α↺α, β↺β and γ↺γ.

Table S3. Structural parameters including cell parameters (a, b), Bond length ($d_{p1\text{-}p2,}$ $d_{p2\text{-}p3)}$, binding energy and band gap of bare CPCS, and two models M_1 and M_2 for α_{PC}^{AA}, α_{PC}^{AB}, α_{PC}^{AC},β_{PC}^{AA}, β_{PC}^{AB}, β_{PC}^{AC}, γ_{PC}^{AA}, γ_{PC}^{AB}, γ_{PC}^{AC},

Figure S1. a) Three starting structures α, β, and γ. b) electron density distributions within α, β, and γ structures.

Figure S2. Three high symmetry stackings AA, AB, and AC for α , β, and γ allotropes

Figure S3. Three hybrid-polytypes of α/β, α /γ, and γ /β

Figure S4. Rotated polytypes are considered including α↺α, β↺β and γ↺γ with rotation angles of 90, 21.79 and 90 degrees for the upper layer

Figure S5. Two models (M_1 and M_2) of arrangement of carbon and phosphorus atoms in two layers of phosphorene carbide

Figure S6. Variation of the Hamiltonian with distance on the basis of the Wannier function

Figure S7. Schematic comparison of layer distance, band gap and binding energy for phosphorene polytypes (α, β, γ, α^{AA}, α^{AB}, α^{AC}, β^{AA}, β^{AB}, β^{AC}, γ^{AA}, γ^{AB}, γ^{AC},α/β, γ /β, α /γ, α↺α, β↺β and γ↺γ). In the absence of gate potential (brown), the lowest gate potential (green) and the highest gate potential (red)

Figure S8. Common Phosphorene Carbide Structure (CPCS) of top (a) and side (b) view.

Figure S9. The structures of homogeneous bilayer α phosphorene Carbide Polytypes from the top and side views in the absence of the gate potential (n_{dop}= 0) and the gate potential (n_{dop}= 2 hole per unit cell).

Figure S10. The structures of homogeneous bilayer β phosphorene carbide polytypes from the top and side views in the absence of the gate potential (n_{dop}= 0) and the gate potential (n_{dop}= 2 hole per unit cell).

Figure S11. The structures of homogeneous bilayer γ phosphorene carbide polytypes from the top and side views in the absence of the gate potential (n_{dop}= 0) and the gate potential (n_{dop}= 2 hole per unit cell)

Figure S12. Structures α^{AA}, α^{AB}, α^{AC}, β^{AA}, β^{AB}, β^{AC}, γ^{AA}, γ^{AB}, γ^{AC} in the absence of gate potential and the highest gate potential (2 hole per unit cell)

Figure S13. The band structure, the CPCS structure in three potentials: 0, 1.5, and 2 hole per unit cell

Figure 14. Sketch of the unit cell along the z-axis, of the difference between the charge density and potential of the doped system ($\rho_{\rm II}^{n}$) and the undoped system $\rho_{\rm II}^{0}$ For a) α^{AA} and b) β^{AA} bilayers.

Table S1. Crystal structure information of the most stable individual phosphorene allotropes.

Structures	Space Group	a (Å)	b (Å)	$d_{p_1-p_2}$	$d_{p_2-p_3}$	d	Number of Atom
α	Orthorhombic	3.31	4.38	2.22	2.28	2.06	4
β	Hexagonal	3.28	3.28	2.27	2.27	1.24	2
	Orthorhombic	3.29	5.70	2.26	2.26	1.24	4
γ	Orthorhombic	3.27	5.42	2.25	2.31	1.49	4

Table S2. Structural parameters including cell parameters (a, b), Bond length ($d_{p1-p2,}$ $d_{p2-p3)}$, thickness of this single layer (d), interlayer distance (D) binding energy and band gap of bare α, β, γ, α^{AA}, α^{AB}, α^{AC}, β^{AA}, β^{AB}, β^{AC}, γ^{AA}, γ^{AB}, γ^{AC}, α/β,α/γ,β/γ and rotated bilayers α↺α, β↺β and γ↺γ.

Structures			Symbol	)Åa (	b (Å)	d_{p1-p2}	d_{p2-p3}	d	D	$E_{Binding}$ (eV)	E_{bg} (eV)
Alpha	monolayer		α	3.31	4.38	2.22	2.26	2.11	-	-3.701	0.91
	bilayer	AA	α^{AA}	3.31	4.45	2.22	2.26	2.13	3.58	-3.722	0.42
		AB	α^{AB}	3.32	4.45	2.22	2.26	2.13	3.22	-3.737	0.45
		AC	α^{AC}	3.31	4.45	2.22	2.26	2.12	3.86	-3.720	0.20
		Rotated	α↺α	13.32	13.36	2.23	2.26	2.2	3.32	-5.492	0.42
Beta	monolayer		β	3.29	5.70	2.26	2.26	1.24	-	-3.713	1.90
	bilayer	AA	β^{AA}	3.29	5.68	2.26	2.26	1.24	3.46	-3.746	1.66
		AB	β^{AB}	3.28	5.68	2.26	2.26	1.24	3.62	-3.736	1.66
		AC	β^{AC}	3.27	5.41	2.26	2.26	1.24	4.38	-3.729	1.60
		Rotated	↺ββ	8.66	8.65	2.27	2.27	1.24	3.63	-5.483	1.48
Gamma	monolayer		γ	3.27	5.42	2.31	2.25	1.49	-	-3.593	0.43
	Bilayer	AA	γ^{AA}	3.42	5.26	2.25	2.28	1.47	2.32	-3.897	0.0
		AB	γ^{AB}	3.27	5.41	2.31	2.25	1.5	3.88	-3.618	0.0
		AC	γ^{AC}	3.27	5.41	2.30	2.25	1.5	3.77	-3.613	0.0
		Rotated	γ↺γ	10.26	10.30	2.23	2.29	1.88	2.30	-5.406	0.0
Hetero bilayer	Alpha-beta		α/β	3.32	17.53	α 2.22	2.25	2.21	3.30	-3.891	0.52
						β 2.27	2.28	1.25			
	Alpha-gamma		α/γ	3.45	21.85	α 2.22	2.25	2.32	3.27	-3.815	0.25
						γ 2.31	2.26	1.52			
	Beta-gamma		γ/β	3.41	5.68	β 2.26	2.24	1.26	3.27	-3.801	0.74
						γ 2.34	2.26	1.49			

Table S3. Structural parameters including cell parameters (a, b), Bond length ($d_{p1\text{-}p2,}$ $d_{p2\text{-}p3)}$), binding energy and band gap of bare CPCS, and two models M_1 and M_2 for α_{PC}^{AA}, α_{PC}^{AB}, α_{PC}^{AC},β_{PC}^{AA}, β_{PC}^{AB}, β_{PC}^{AC}, γ_{PC}^{AA}, γ_{PC}^{AB}, γ_{PC}^{AC}.

Structures			symbol	a (Å)	b (Å)	$d_{p_1-p_2}$	$d_{p_2-p_3}$	$E_{Binding}$ (eV)	E_{bg} (eV)
Common Phosphorene carbide structure			CPCS	2.91	5.09	1.74	1.76	-4.788	0.0
αC bilayer	AA	M_1	$\alpha_{PC-m_1}^{AA}$	2.90	4.21	1.78	1.71	-4.899	0.62
		M_2	$\alpha_{PC-m_2}^{AA}$	2.90	4.91	1.74	1.73	-4.871	0.0
	AB	M_1	$\alpha_{PC-m_1}^{AB}$	2.89	4.92	1.74	1.73	-4.773	0.0
		M_2	$\alpha_{PC-m_2}^{AB}$	2.95	5.04	1.75	1.76	-5.015	0.88
	AC	M_1	$\alpha_{PC-m_1}^{AC}$	2.94	4.60	1.80	1.69	-4.846	0.19
		M_2	$\alpha_{PC-m_2}^{AC}$	2.94	4.67	1.80	1.68	-4.993	0.86
βC bilayer	AA	M_1	$\beta_{PC-m_1}^{AA}$	2.95	5.11	1.78	1.78	-4.727	0.0
		M_2	$\beta_{PC-m_2}^{AA}$	2.94	5.10	1.77	1.77	-4.714	0.0
	AB	M_1	$\beta_{PC-m_1}^{AB}$	2.95	5.11	1.80	1.80	-4.740	0.0
		M_2	$\beta_{PC-m_2}^{AB}$	2.95	5.11	1.78	1.78	-4.725	0.0
	AC	M_1	$\beta_{PC-m_1}^{AC}$	2.95	5.11	1.91	1.91	-5.176	0.0
		M_2	$\beta_{PC-m_2}^{AC}$	2.90	5.02	1.88	1.88	-5.038	1.59
γC bilayer	AA	M_1	$\gamma_{PC-m_1}^{AA}$	2.92	5.06	1.76	1.76	-4.975	0.31
		M_2	$\gamma_{PC-m_2}^{AA}$	2.93	4.71	1.90	1.80	-4.995	0.87
	AB	M_1	$\gamma_{PC-m_1}^{AB}$	2.91	4.80	1.83	1.91	-5.047	0.94
		M_2	$\gamma_{PC-m_2}^{AB}$	2.92	5.01	1.78	1.74	-4.932	0.0
	AC	M_1	$\gamma_{PC-m_1}^{AC}$	2.92	5.06	1.76	1.76	-4.975	0.31
		M_2	$\gamma_{PC-m_2}^{AC}$	2.89	4.97	1.83	1.88	-5.046	0.68

Figure S1. a) Three starting structures α, β, and γ. b) electron density distributions within α, β, and γ structures.

(a)

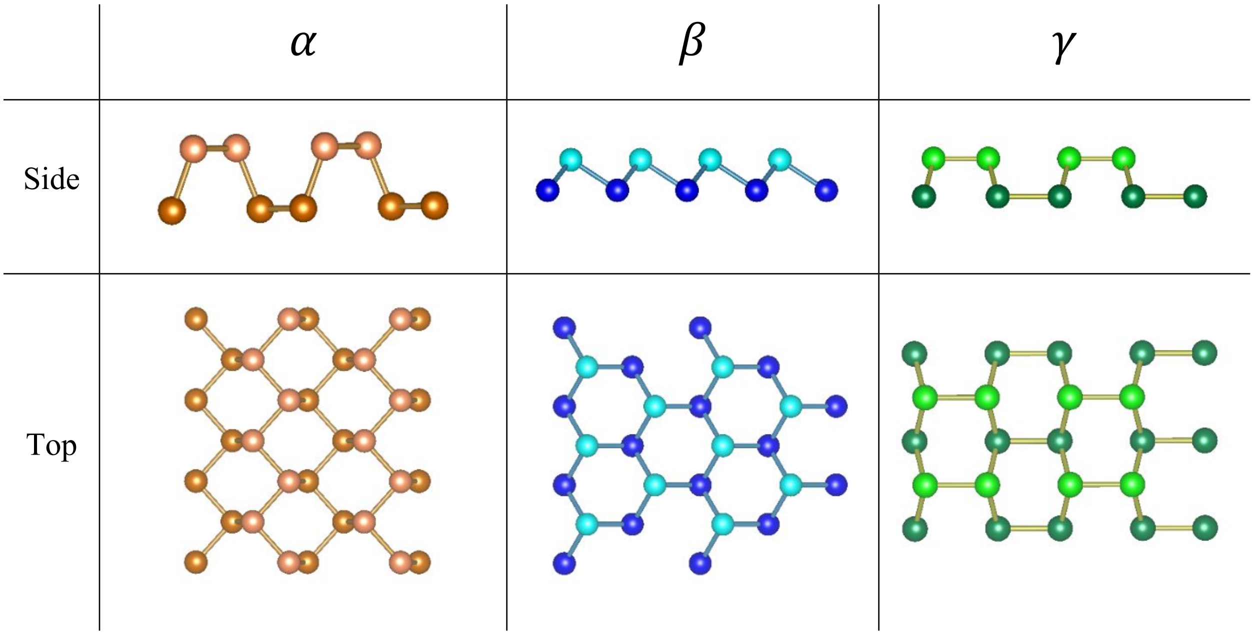

(b)

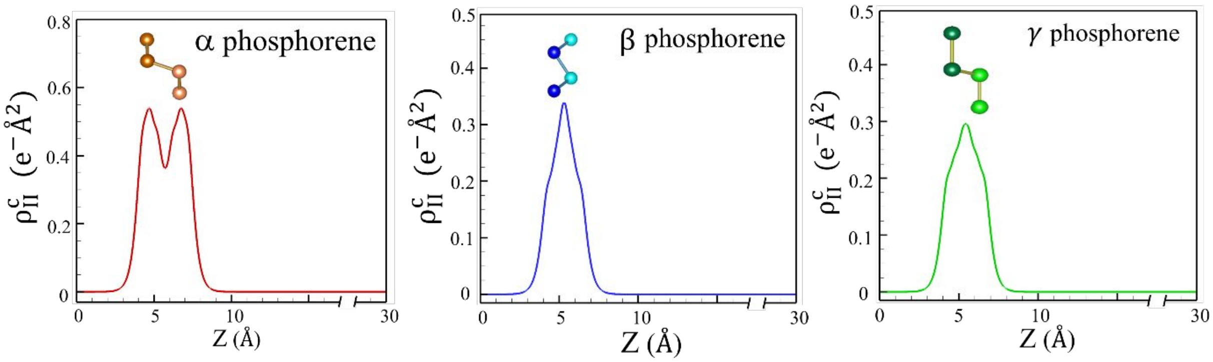

Figure S2. Three high symmetry stackings AA, AB, and AC for α , β, and γ allotropes.

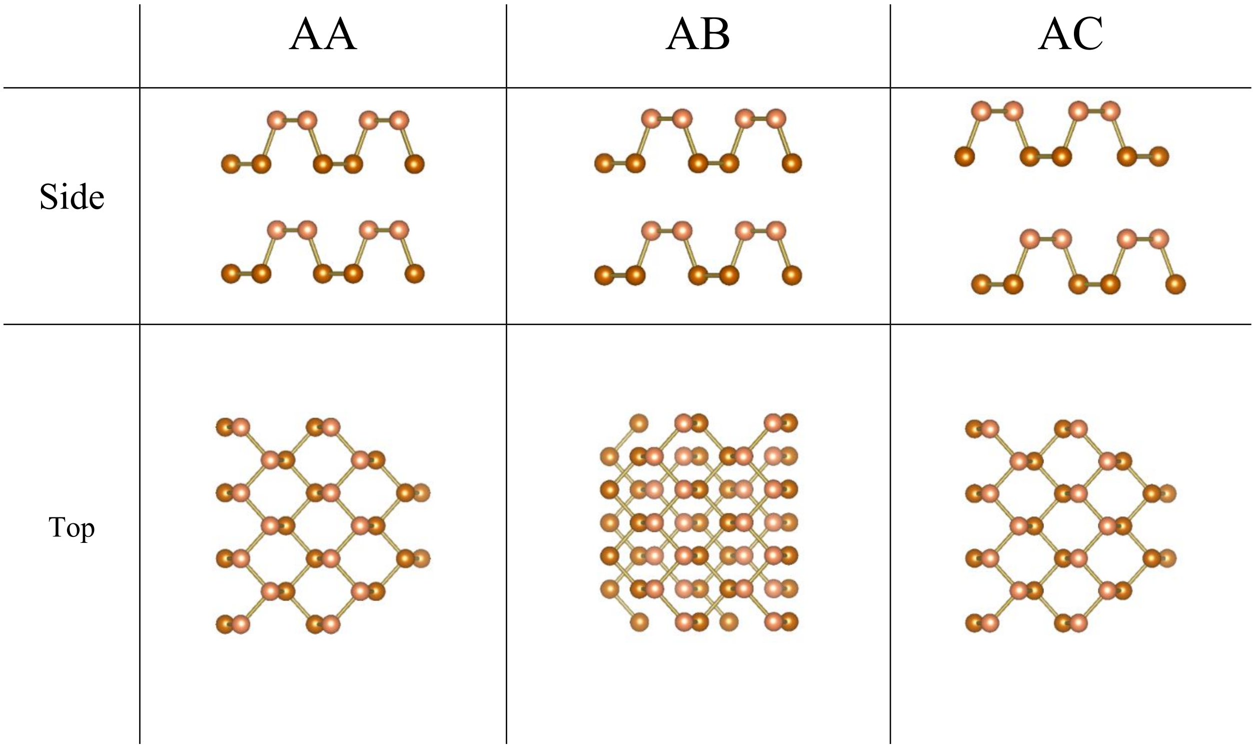

Figure S3. Three hybrid-polytypes of α/β, α /γ, and γ /β.

α/β	α /γ	γ/β
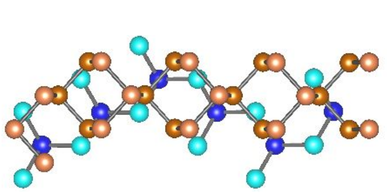	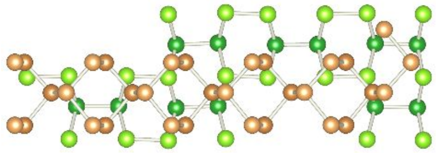	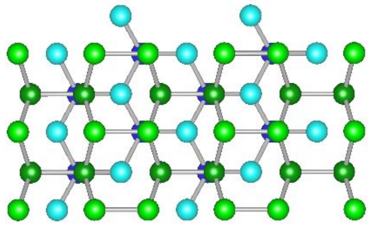
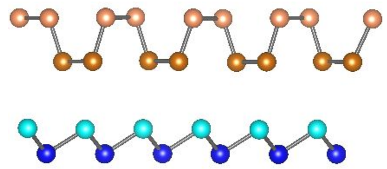	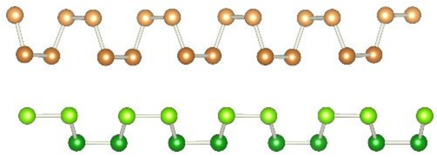	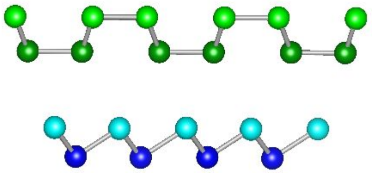

Figure S4. Rotated polytypes are considered including α↺α, β↺β and γ↺γ with rotation angles of 90, 21.79 and 90 degrees for the upper layer

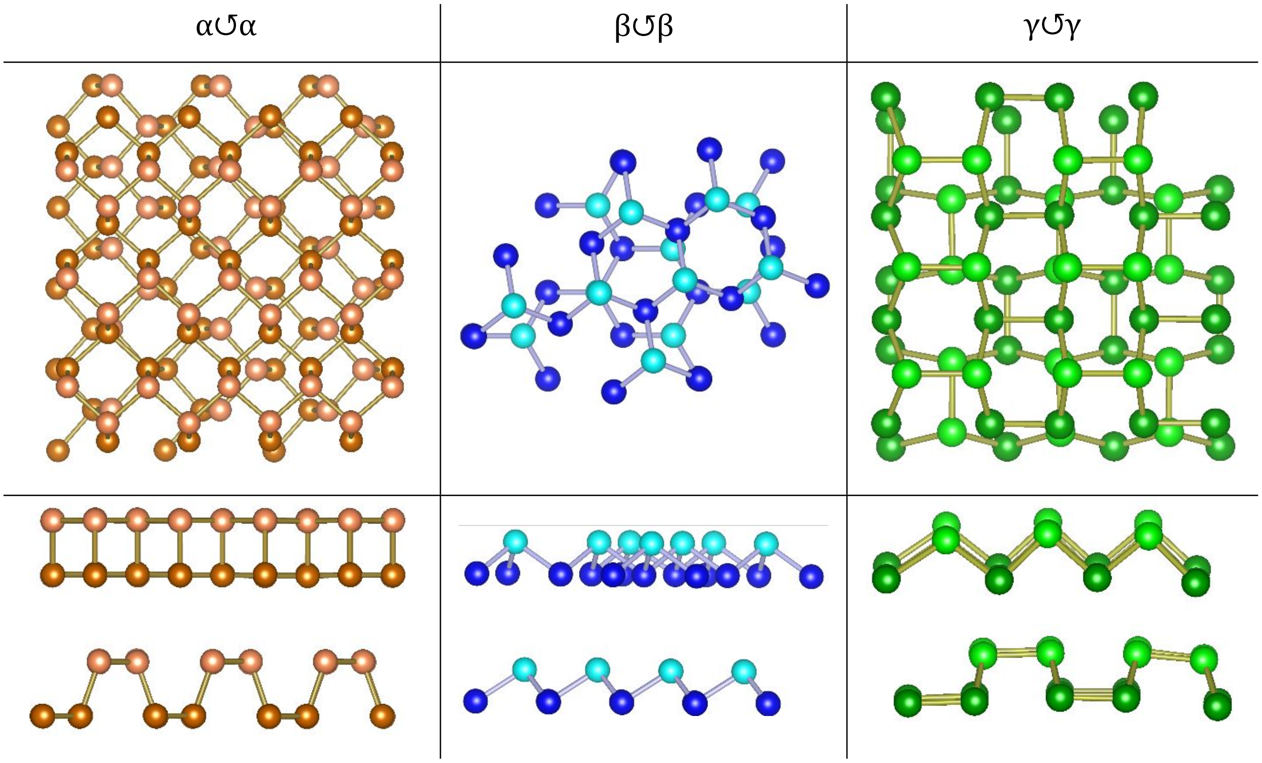

Figure S5. Two models (M_1 and M_2) of arrangement of carbon and phosphorus atoms in two layers of phosphorene carbide

	M_1	M_2
Side	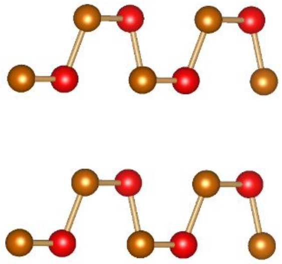	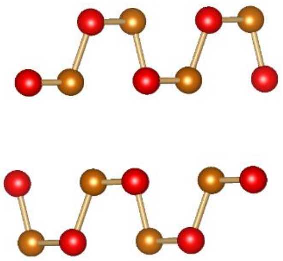
Top	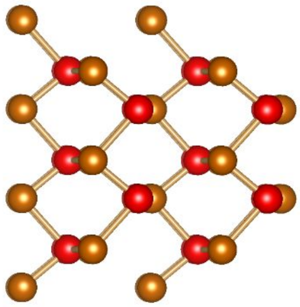	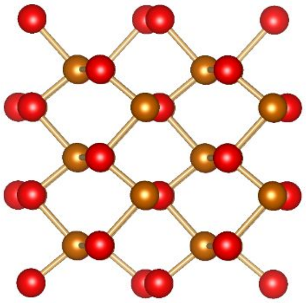

Figure S6. Variation of the Hamiltonian with distance on the basis of the Wannier function

CPCS	β	Phosphorene	Graphene
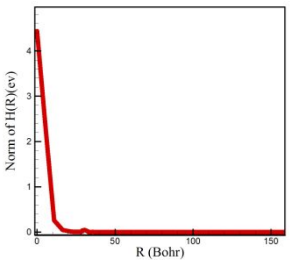	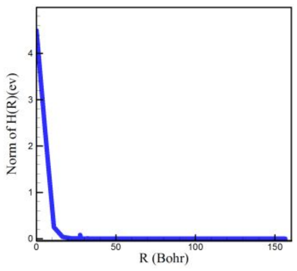	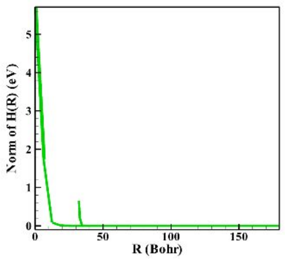	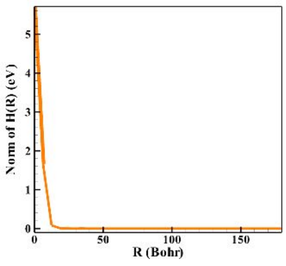

Figure S7. Schematic comparison of layer distance, band gap and binding energy for phosphorene polytypes (α, β, γ, α^{AA}, α^{AB}, α^{AC}, β^{AA}, β^{AB}, β^{AC}, γ^{AA}, γ^{AB}, γ^{AC},α/β, γ /β, α /γ, α↺α, β↺β and γ↺γ). In the absence of gate potential (brown), the lowest gate potential (green) and the highest gate potential (red)

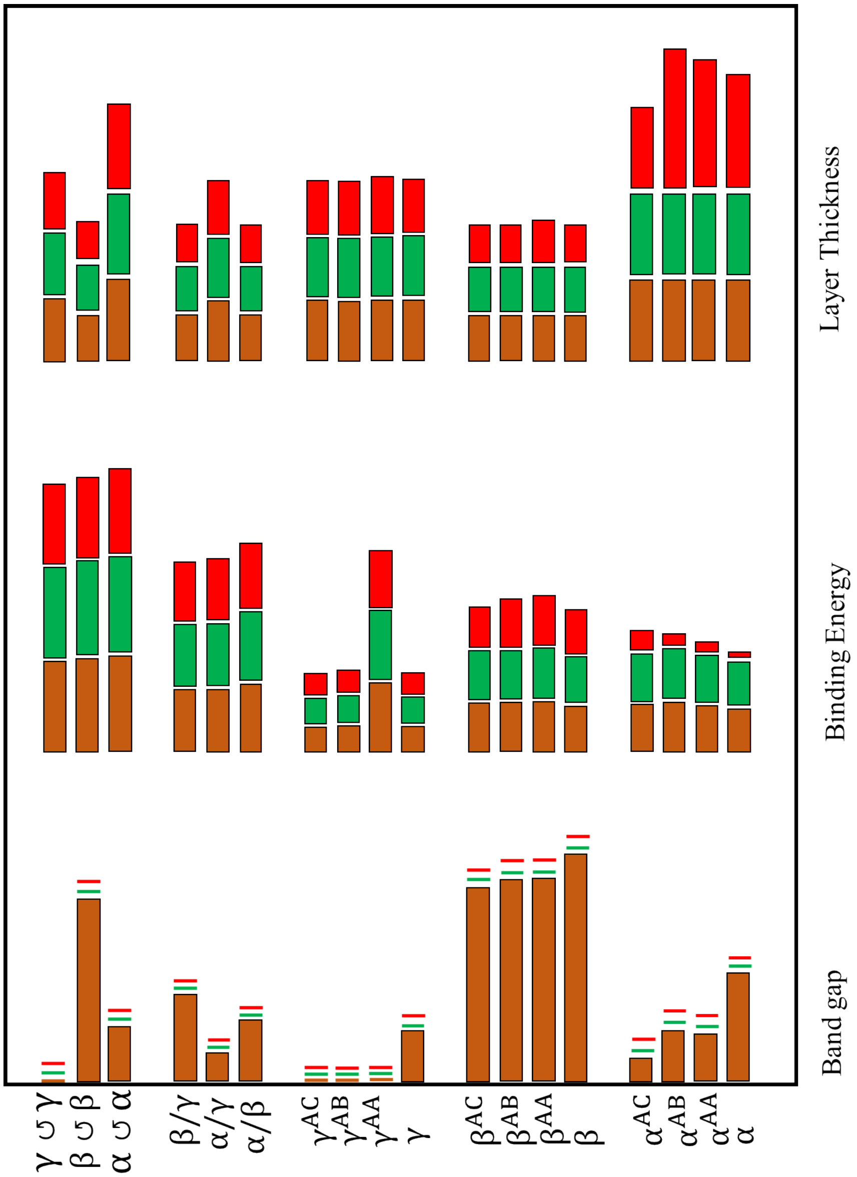

Figure S8. Common Phosphorene Carbide Structure (CPCS) of top (a) and side (b) view.

a)

b)

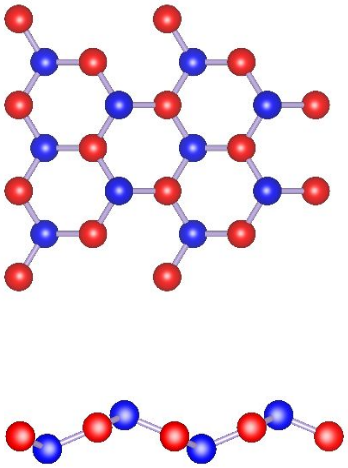

Figure S9. The structures of homogeneous bilayer α phosphorene Carbide Polytypes from the top and side views in the absence of the gate potential (n_{dop}= 0) and the gate potential (n_{dop}= 2 hole per unit cell).

$\alpha_{PC}^{AA} - m_1$

	n_{dop}= 0	n_{dop}= 2
Side	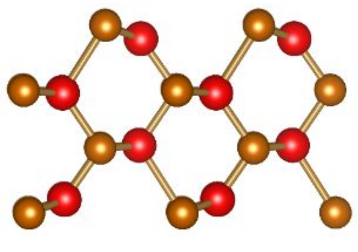	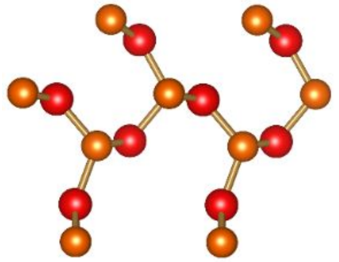
Top	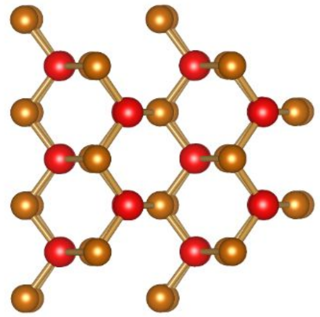	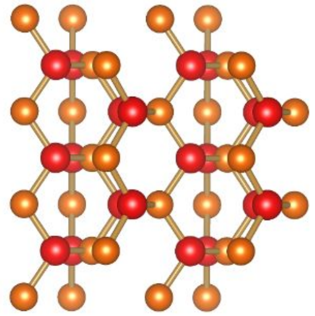

$\alpha_{PC}^{AB} - m_1$

	n_{dop}= 0	n_{dop}= 2
Side	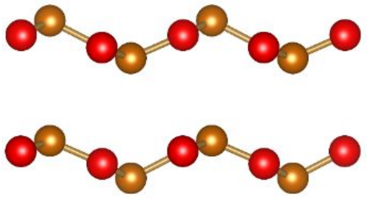	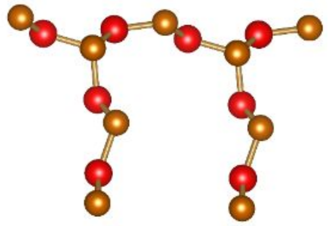

Top	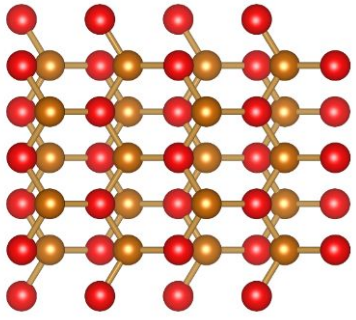	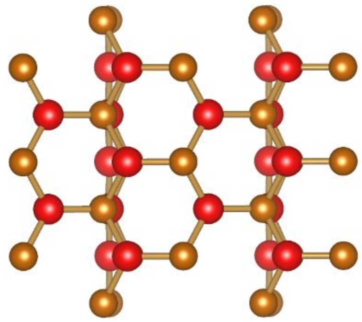

$\alpha_{PC-m_1}^{AC}$

	n_{dop}= 0	n_{dop}= 2
Side	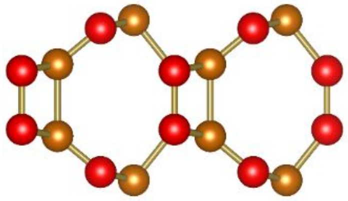	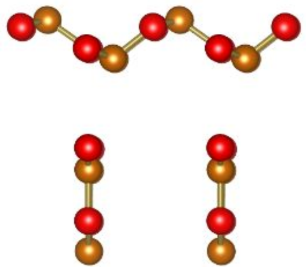
Top	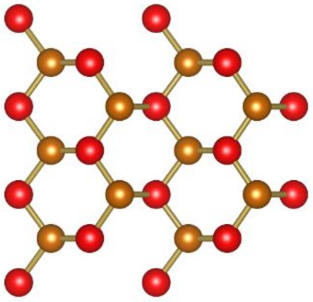	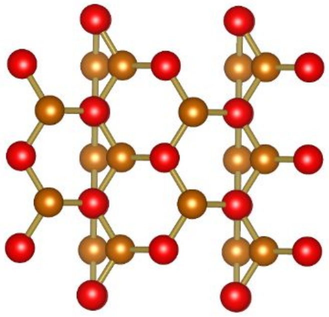

$\alpha_{PC-m_2}^{AA}$

	n_{dop}= 0	n_{dop}= 2
Side	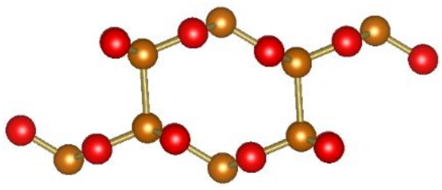	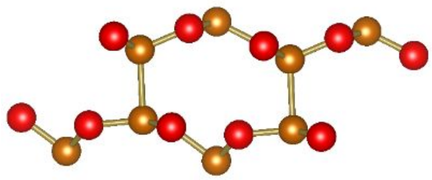

Top

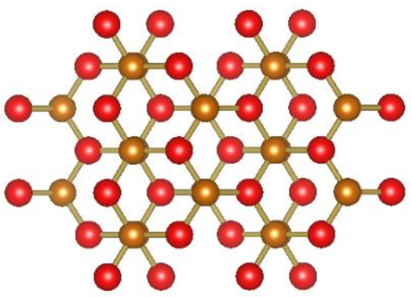

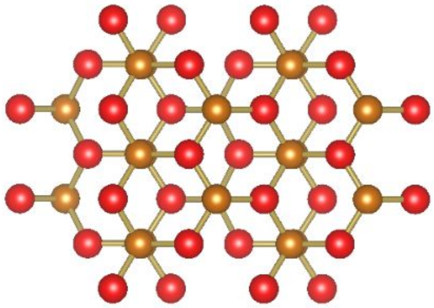

$\alpha_{PC-m_2}^{AB}$

	n_{dop}= 0	n_{dop}= 2
Side	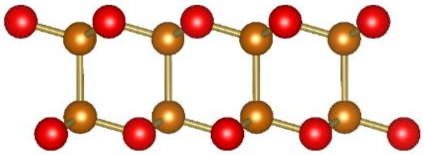	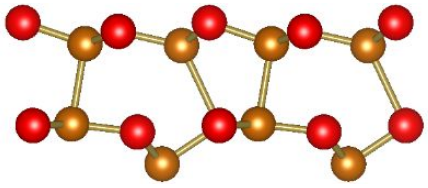
Top	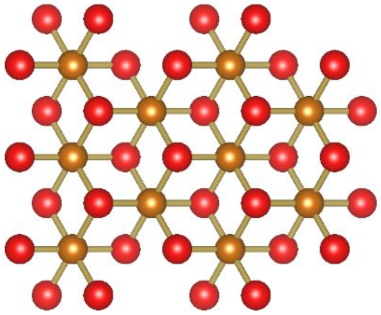	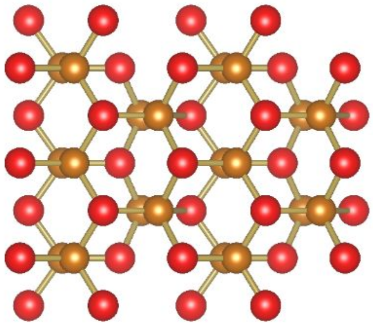

$\alpha_{PC-m_2}^{AC}$

	n_{dop}= 0	n_{dop}= 2
	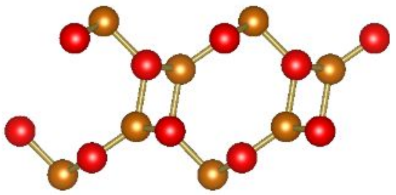	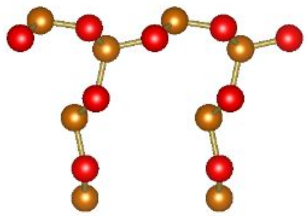
	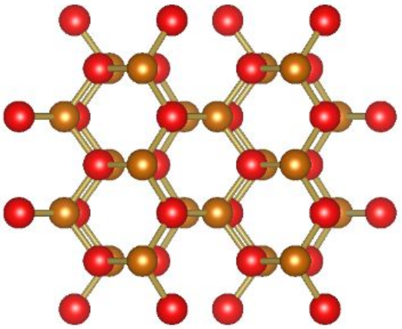	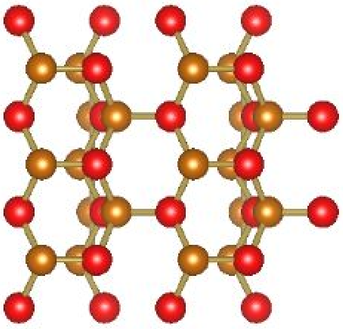

Figure S10. The structures of homogeneous bilayer β phosphorene carbide polytypes from the top and side views in the absence of the gate potential (n_{dop}= 0) and the gate potential (n_{dop}= 2 hole per unit cell).

$\beta_{PC}^{AA} - m1$

	n_{dop}= 0	n_{dop}= 2
Side	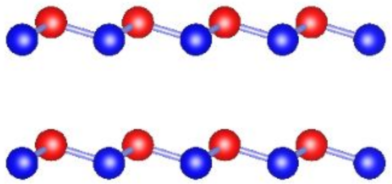	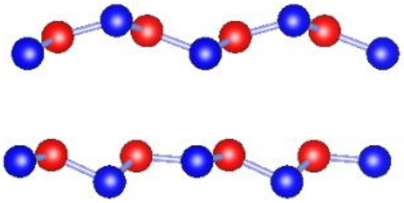
Top	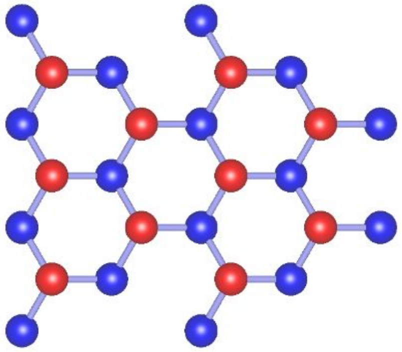	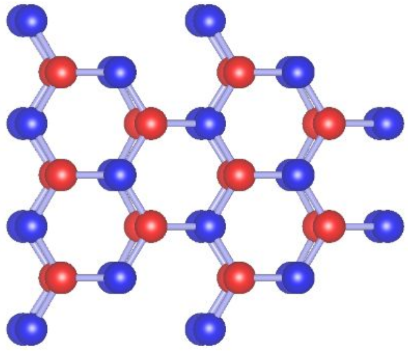

$\beta_{PC}^{AB} - m1$

	n_{dop}= 0	n_{dop}= 2
Side	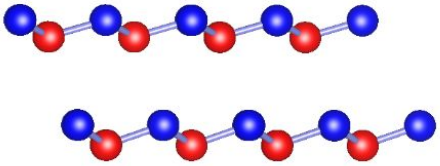	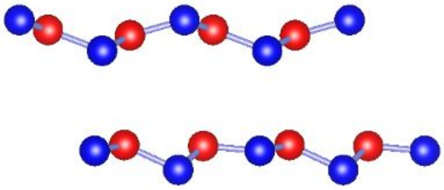
Top	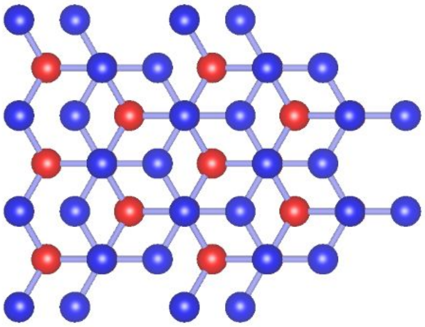	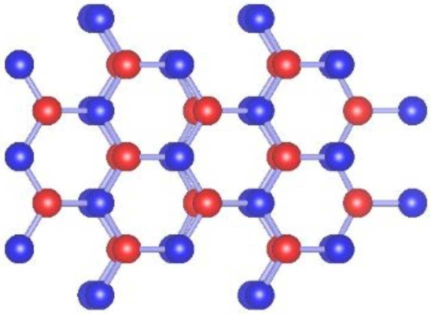

$\beta^{\mathrm{AC}}_{\mathrm{PC-m1}}$

	n_{dop}= 0	n_{dop}= 2
Side	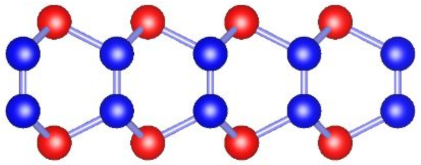	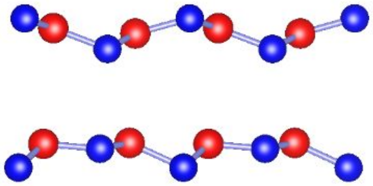
Top	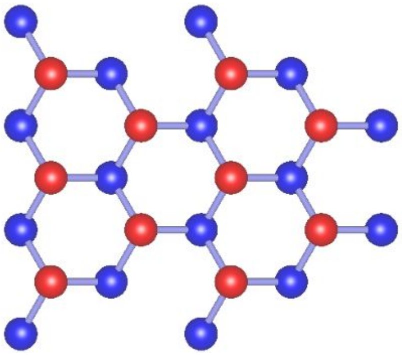	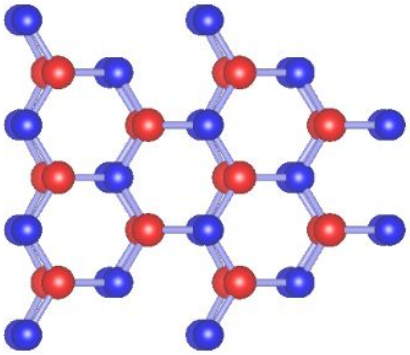

$\beta^{\mathrm{AA}}_{\mathrm{PC-m2}}$

	n_{dop}= 0	n_{dop}= 2
Side	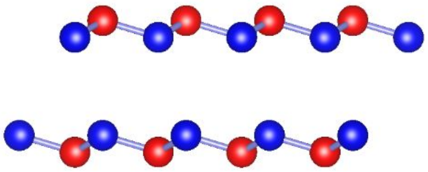	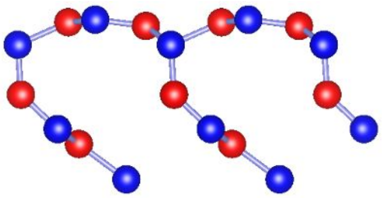
Top	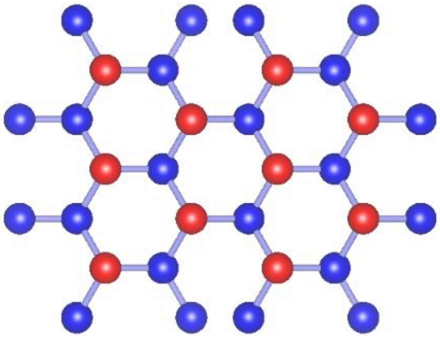	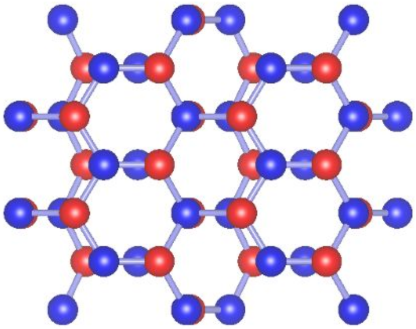

$\beta^{\mathrm{AB}}_{\mathrm{PC-m2}}$

	n_{dop}= 0	n_{dop}= 2
Side	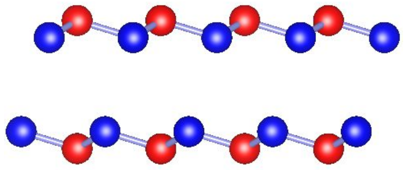	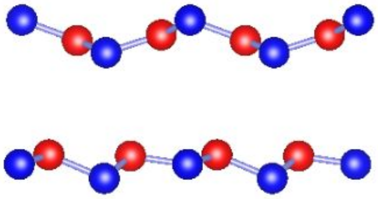
Top	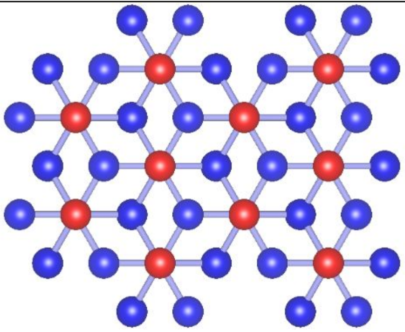	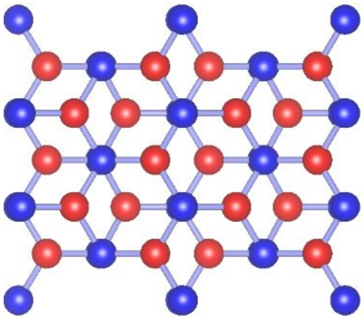

$\beta^{\mathrm{AC}}_{\mathrm{PC-m2}}$

	n_{dop}= 0	n_{dop}= 2
Side	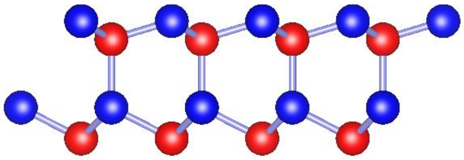	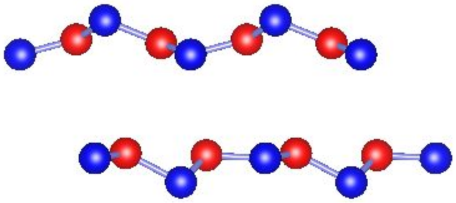
Top	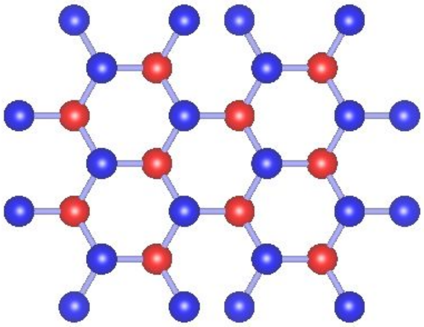	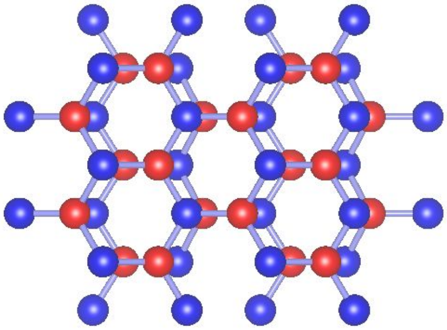

Figure S11. The structures of homogeneous bilayer γ phosphorene carbide polytypes from the top and side views in the absence of the gate potential (n_{dop}= 0) and the gate potential (n_{dop}= 2 hole per unit cell)

$\gamma_{PC}^{AA}-m1$

	n_{dop}= 0	n_{dop}= 2
Side	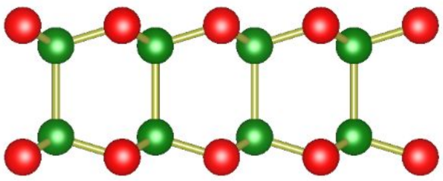	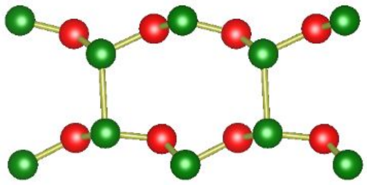
Top	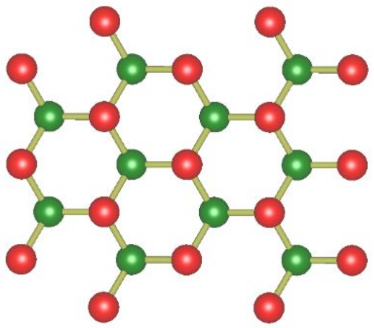	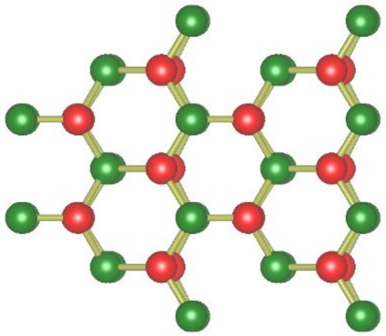

$\gamma_{PC}^{AB}-m1$

	n_{dop}= 0	n_{dop}= 2
Side	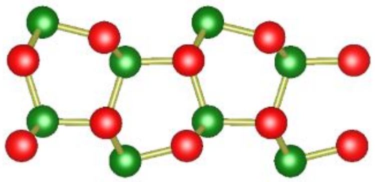	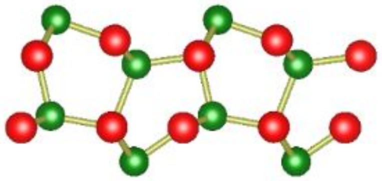

Top

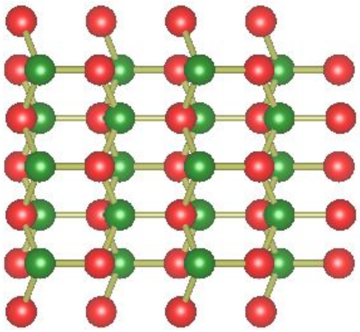

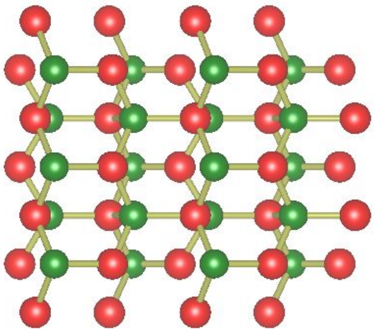

γ^{AC}_{PC-m1}

	n_{dop}= 0	n_{dop}= 2
Side	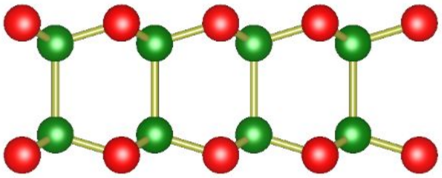	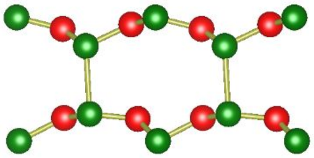
Top	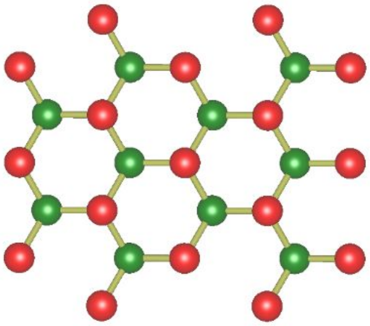	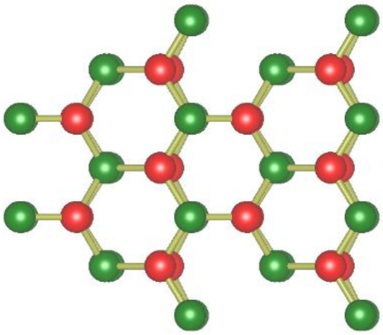

γ^{AA}_{PC-m2}

	n_{dop}= 0	n_{dop}= 2
Side	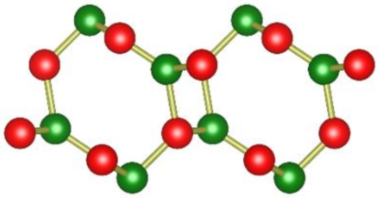	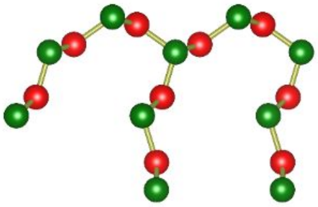
Top	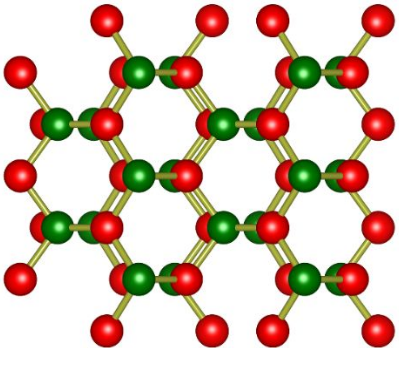	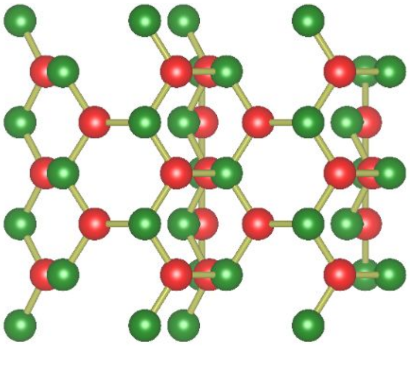

γ_{PC-m2}^{AB}

	n_{dop}= 0	n_{dop}= 2
Side	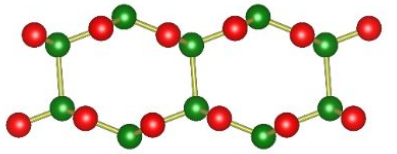	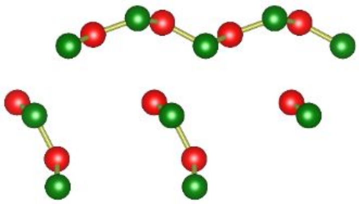
Top	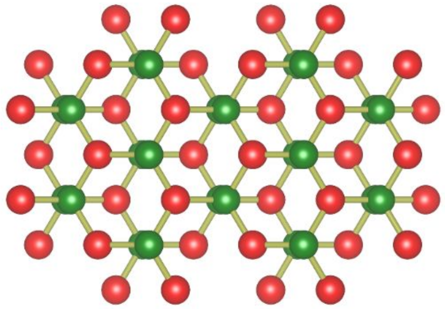	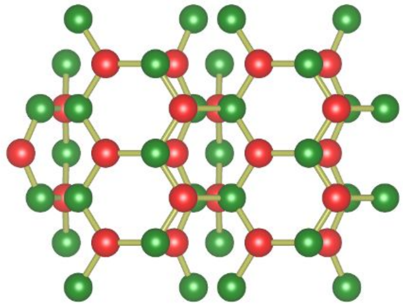

γ_{PC-m2}^{AC}

	n_{dop}= 0	n_{dop}= 2
Side	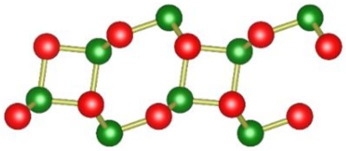	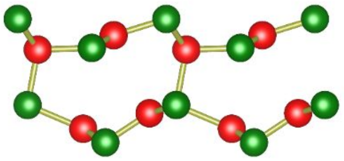
Top	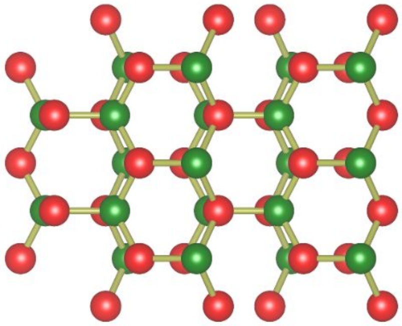	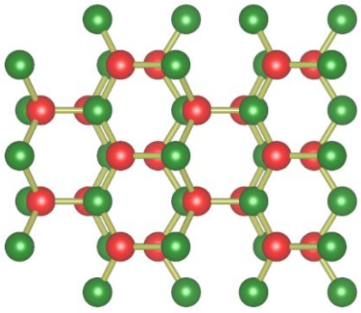

Figure S12. Structures α^{AA}, α^{AB}, α^{AC}, β^{AA}, β^{AB}, β^{AC}, γ^{AA}, γ^{AB}, γ^{AC} in the absence of gate potential and the highest gate potential (2 hole per unit cell)

α^{AA}

	n_{dop}= 0	n_{dop}= 2
Side	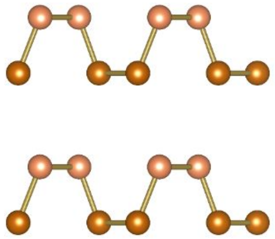	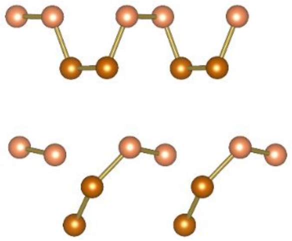
Top	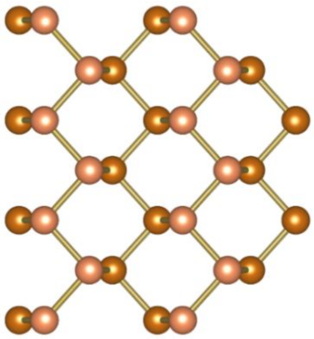	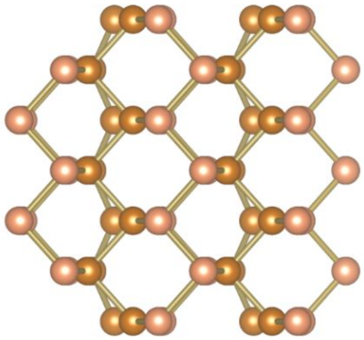

α^{AB}

	n_{dop}= 0	n_{dop}= 2
Side	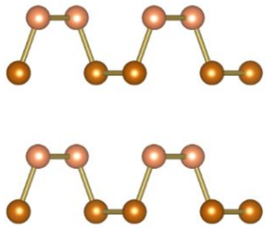	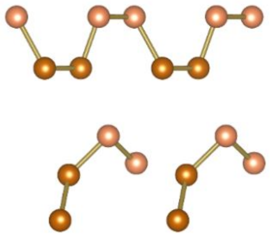

Top	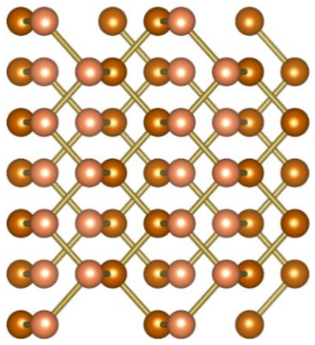	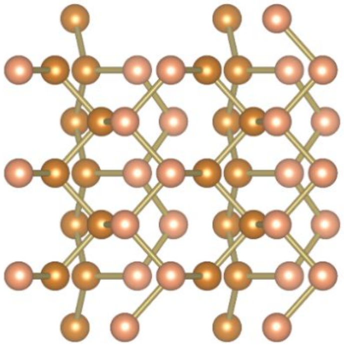
α^{AC}		
	n_{dop}= 0	n_{dop}= 2
Side	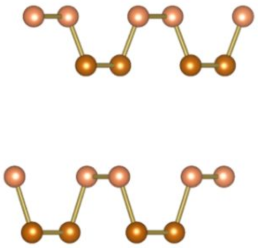	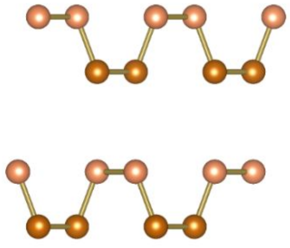
Top	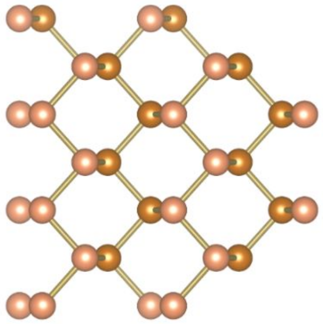	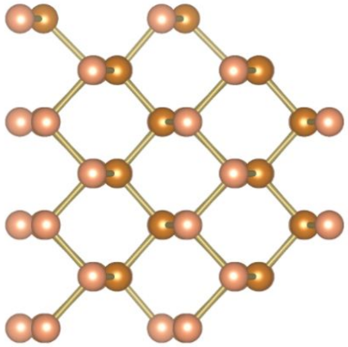
β^{AA}		
	n_{dop}= 0	n_{dop}= 2
Side	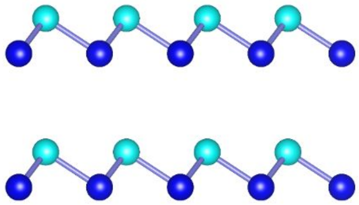	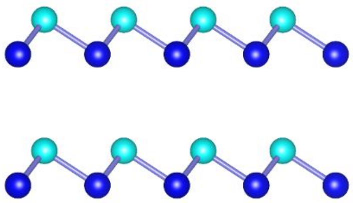

Top	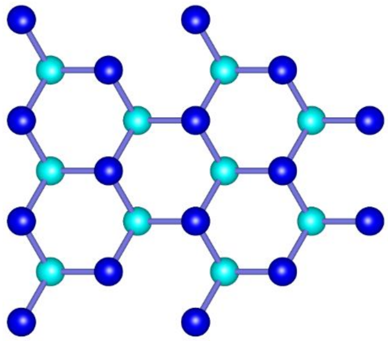	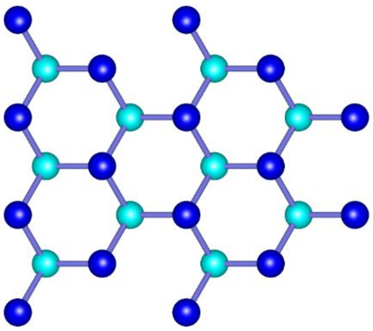

β^{AB}

	n_{dop}= 0	n_{dop}= 2
Side	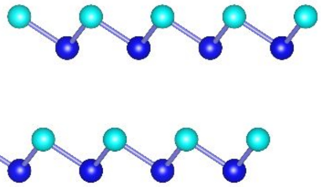	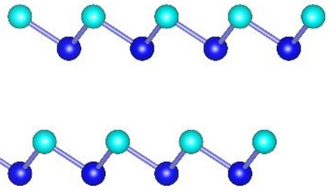
Top	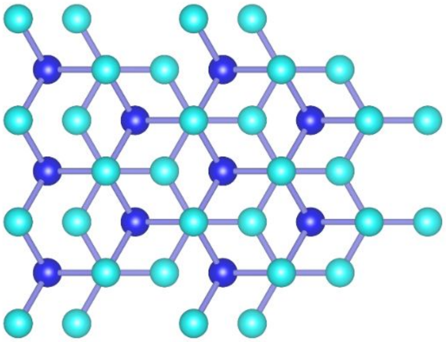	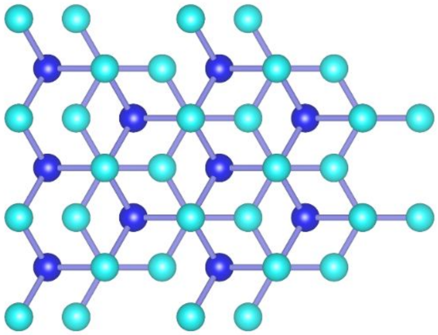

β^{AC}

	n_{dop}= 0	n_{dop}= 2
Side	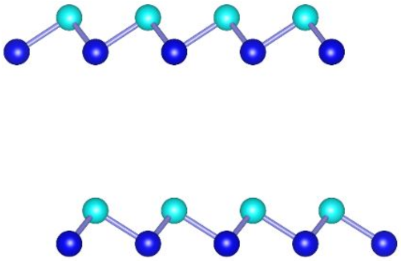	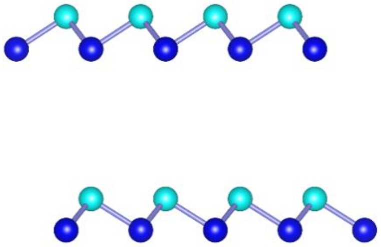

Top	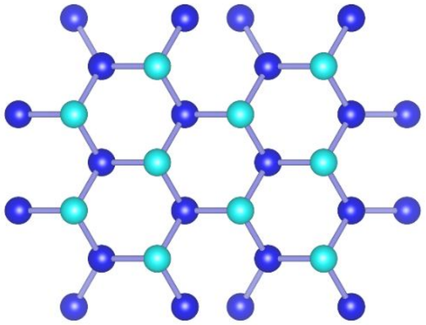	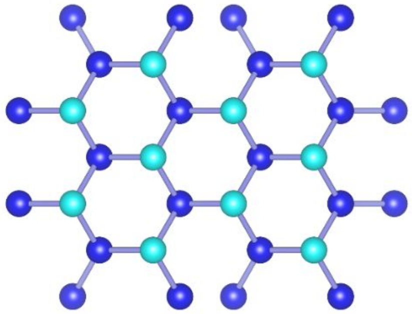
	γ^{AA}	
	n_{dop}= 0	n_{dop}= 2
Side	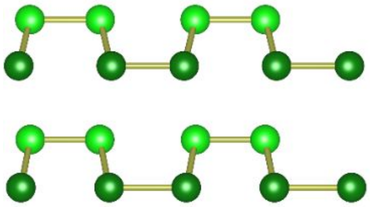	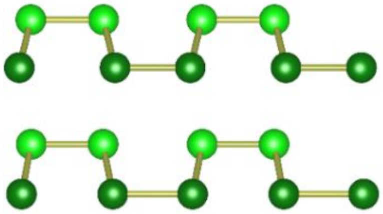
Top	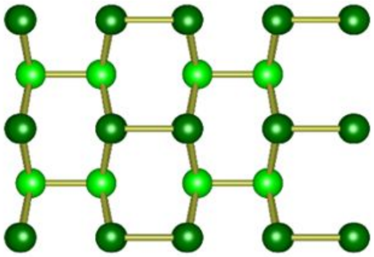	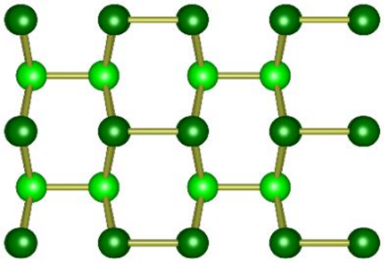
	γ^{AB}	
	n_{dop}= 0	n_{dop}= 2
Side	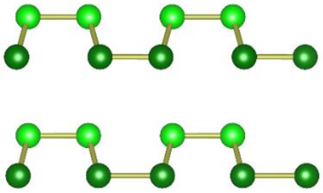	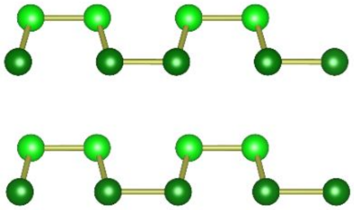

Top	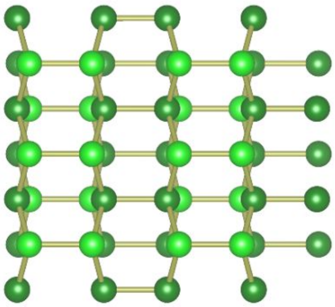	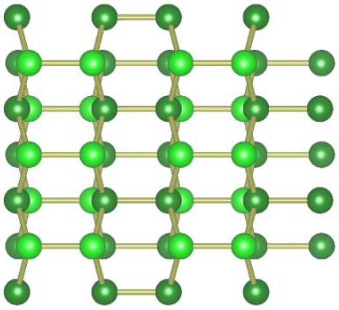
γ^{AC}		
	n_{dop}= 0	n_{dop}= 2
Side	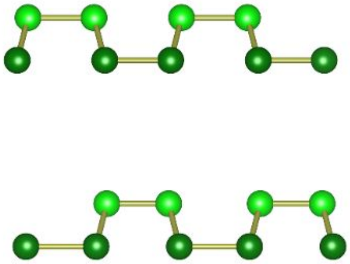	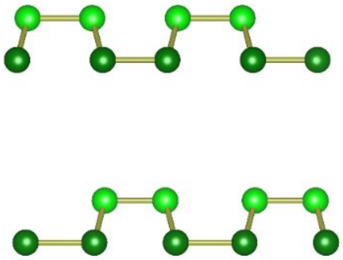
Top	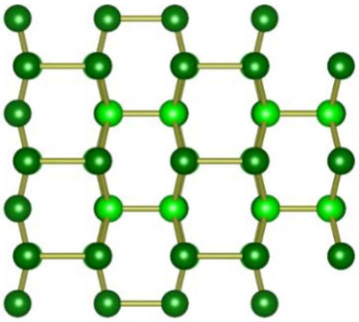	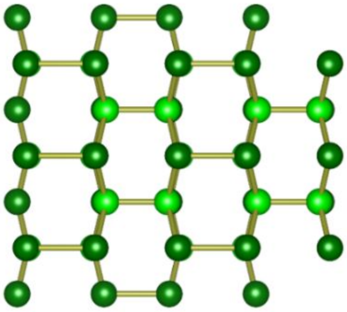

Figure S13. The band structure, the CPCS structure in three potentials: 0, 1.5, and 2 hole per unit cell

	n_{dop}= 0	n_{dop}= 1.5	n_{dop}= 2.0
CPCS	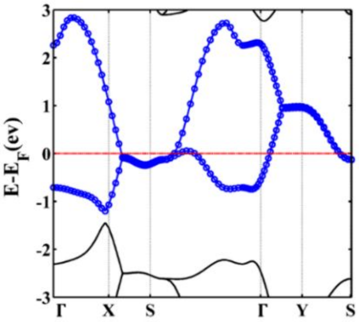	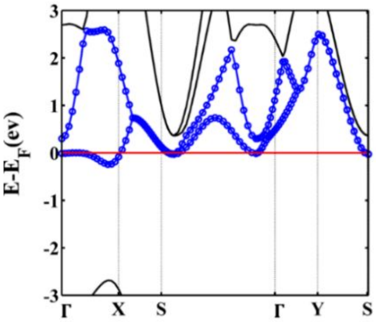	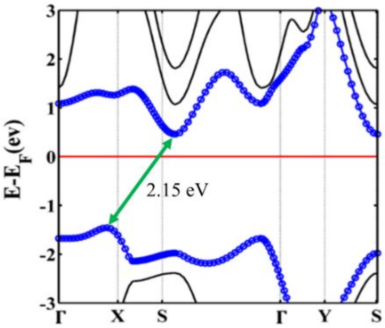

Figure 14. Sketch of the unit cell along the z-axis, of the difference between the charge density and potential of the doped system (ρ_{II}^{n}) and the undoped system ρ_{II}^{0} For a) α^{AA} and b) β^{AA} bilayers.

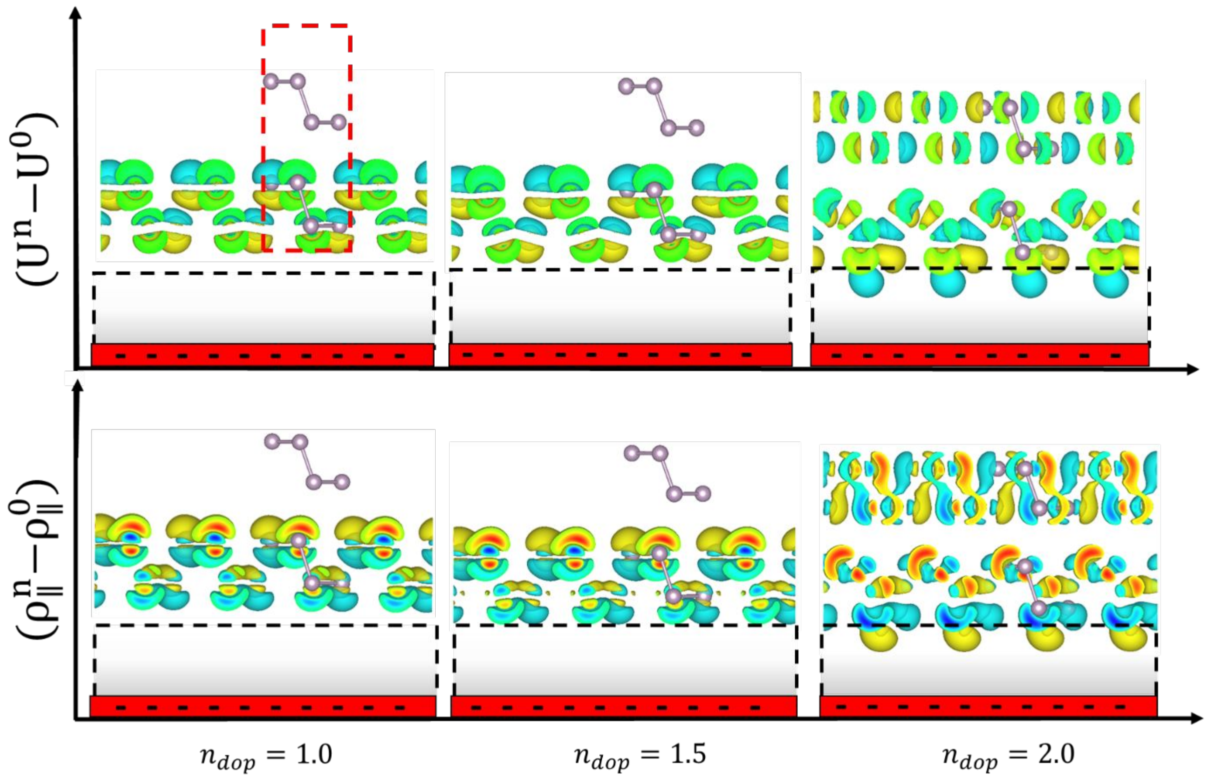

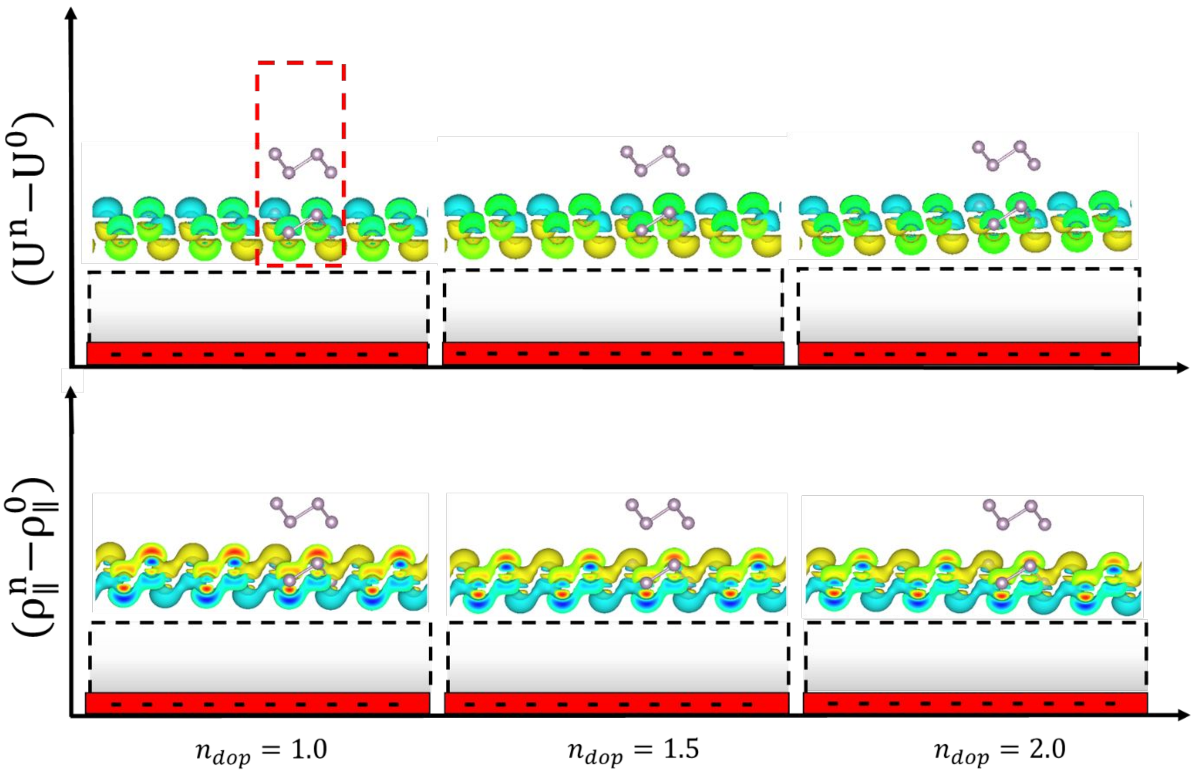

$(U^n - U^0)$
$(\rho_{\parallel}^{n} - \rho_{\parallel}^{0})$
$n_{dop} = 1.0$
$n_{dop} = 1.5$
$n_{dop} = 2.0$
